# Shape morphing of plastic films

**DOI:** 10.1038/s41467-022-34844-y

**Published:** 2022-11-26

**Authors:** Feilong Zhang, Dong Li, Changxian Wang, Zhihua Liu, Man Yang, Zequn Cui, Junqi Yi, Ming Wang, Ying Jiang, Zhisheng Lv, Shutao Wang, Huajian Gao, Xiaodong Chen

**Affiliations:** 1 Innovative Center for Flexible Devices (iFLEX), Max Planck–NTU Joint Lab for Artificial Senses, School of Materials Science and Engineering, Nanyang Technological University, 50 Nanyang Avenue, Singapore, 639798 Singapore; 2 School of Mechanical and Aerospace Engineering, Nanyang Technological University, 50 Nanyang Avenue, Singapore, 639798 Singapore; 3 Institute of Materials Research and Engineering (IMRE), Agency for Science Technology and Research, 2 Fusionopolis Way, Innovis, #08-03, Singapore, 138634 Singapore; 4 CAS Key Laboratory of Bio-inspired Materials and Interfacial Science, CAS Center for Excellence in Nanoscience, Technical Institute of Physics and Chemistry, Chinese Academy of Sciences, Beijing, 100190 P. R. China; 5 Institute of High-Performance Computing, Agency for Science Technology and Research, 1 Fusionopolis Way, #16-16 Connexis, Singapore, 138632 Singapore

**Keywords:** Mechanical properties, Mechanical engineering

## Abstract

Three-dimensional (3D) architectures have qualitatively expanded the functions of materials and flexible electronics. However, current fabrication techniques for devices constrain their substrates to 2D geometries and current post-shape transformation strategies are limited to heterogenous or responsive materials and are not amenable to free-standing inert plastic films such as polyethylene terephthalate (PET) and polyimide (PI), which are vital substrates for flexible electronics. Here, we realize the shape morphing of homogeneous plastic films for various free-standing 3D frameworks from their 2D precursors by introducing a general strategy based on programming the plastic strain in films under peeling. By modulating the peeling parameters, previously inaccessible free-standing 3D geometries ranging from millimeter to micrometer were predicted theoretically and obtained experimentally. This strategy is applicable to most materials capable of plastic deformation, including polymers, metals, and composite materials, and can even enable 4D transformation with responsive plastic films. Enhanced performance of 3D circuits and piezoelectric systems demonstrates the enormous potential of peeling-induced shape morphing for 3D devices.

## Abstract (web summary)

Shape morphing offers a promising method to transform 2D electronics into 3D structured devices. Here authors show a shape morphing strategy of homogeneous inert plastic thin films into free-standing 3D frameworks by programming the plastic strain in films under peeling.

## Introduction

Three-dimensional (3D) architectures are highly desired for functional materials and electronic devices because they can accommodate more functions^1,2^ and offer better spatial resolution than those restricted to two-dimensional (2D) architectures^3–7^. For example, 2D strain sensors can monitor only the magnitudes of in-plane forces^8,9^, while a 3D strain sensor recognizes both the magnitude and directions of out-of-plane forces^4^. Similarly, 2D photodetectors measure only intensity, while 3D photodetectors can simultaneously sense both the intensity and direction of illumination^7^. Unfortunately, methods to directly produce these 3D devices are limited, because most existing fabrication technologies, such as spin coating^10^, lithographic patterning^11^, etching, and thin film deposition^12^ constrain the substrates of devices to 2D or only simple macroscale 3D geometries.

Shape morphing offers a promising method to transform 2D electronics into 3D structured devices^2,3,13,14^. The method relies on the inner stress caused by heterogenous expansion or compression of responsive materials^15^. Using responsive materials, shape morphing has been achieved in artificial materials with heterogeneous components or structures such as the bending or twisting of responsive bilayer polymers^16,17^, patterned polymers^18^, liquid crystal polymer^19^, and polymer films with gradient structures^20^, for applications in simple 3D electrodes^21,22^. More recently, compressive buckling was employed to transform 2D micro/nanostructures into 3D structured devices fixed on contractive substrates^2,3,23,24^. These methods are useful for creating 3D architectures; however, they are either limited to heterogeneous or bilayer-responsive materials, or inaccessible to free-standing 3D architectures of inert homogenous plastic materials such as polyethylene terephthalate (PET), polyimide (PI), and polytetrafluoroethylene (PTFE), which are insensitive to external stimuli and cannot easily change shapes. Because these plastic films are common and important substrates for current flexible thin film devices^12,22^, realizing the shape morphing of these materials will offer a simple and direct method to produce a variety of functional and flexible 3D devices for various applications.

Here, we report a new strategy in which mechanically peeling 2D plastic film precursors from adhesive substrates can program the distribution and direction of the plastic strains in the films to produce a variety of free-standing 3D frameworks. Mechanical peeling models were built to reveal the mechanism of shape morphing and predict the morphologies after peeling. By tuning the peeling parameters such as adhesion energy, peeling speed, and peeling direction, we were able to tune the global or local curvature of the peeled film to obtain tubes, helices, spirals, polygons, hyperboloids, and other complicated shapes. When the plastic films were used as substrates, peeling-induced 3D electronics (circuits and piezoelectric systems) and custom-shaped elastomer films were also achieved. The proposed peeling-induced shape morphing is applicable to most materials capable of plastic deformation and other complex systems like plastic-elastomer bilayer films with responsive materials for 4D structures (i.e., responsive 3D structures), which could open new avenues in 3D and 4D devices.

## Results

### Peeling model and peeling-induced asymmetric plastic strains

**Fig. 1:**
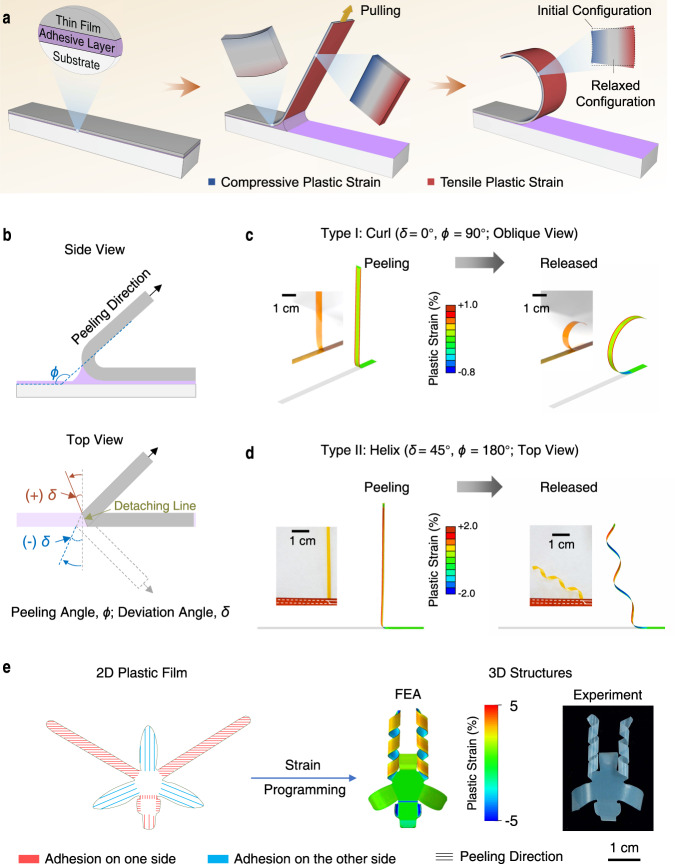
Mechanism of peeling-induced shape morphing of plastic films. **a** Schematic showing the peeling process involving the adherent thin film, adhesive layer, and substrate. The thin film is bent at the detaching area causing asymmetric deformation (tension and compression) on the two sides. The asymmetric plastic strain remains in the film after releasing and leads to the bending of the film. **b** Schematic showing two important control parameters: the peeling angle, *ϕ*, indicates the supplementary dihedral angle between the detached part and adhered part of the plastic film; the deviation angle, *δ*, indicates the angle measured from the short axis of the adherent film to the detaching line. According to the definition of peeling directions, the peeling process can be divided into two categories: *δ* = 0° and *δ* ≠ 0°. **c**, **d** Experimental and FEA results showing the peeling of a plastic PI film (30 μm thick, 5 mm wide) from Kapton tape at *ϕ* = 90° and *δ* = 0° (**c**) and *ϕ* = 180° and *δ* = 45° (with peeling force perpendicular to the long axis of the film) (**d**) during peeling (left) and after release (right). Peeling induces plastic deformation in PI and results in curling at *δ* = 0° and helix at *δ* = 45°. Insets: photographs of plastic PI film during and after peeling, respectively. **e** An orchid-like architecture with configurations of bending and helix from a 2D precursor film by peeling. Colors in the 3D FEA indicate the maximum principal plastic strain.

Peeling is a common method to detach materials from substrates. An example is the removal of adhesive tape from surfaces^25^. It was discovered piecemeal that plastic yielding during peeling can cause inert adherend plastic films to curl^26^, which can be taken as a clue for potential shape morphing of inert plastic films. The peeling system includes the adherent film, adhesive layer, and substrate (Fig. 1a, left), where asymmetric plastic strains are induced at the two sides of the film after peeling (Fig. 1a, right). The extent and direction of peeling-induced deformation for a film belt were mainly controlled by two parameters: the peeling angle *ϕ* (the supplementary dihedral angle between the detached part and adhered part of the plastic film) (Fig. 1b, top) and the deviation angle *δ* (defined as the rotation angle measured from the short axis of the adhered film belt to the detaching line) (Fig. 1b, bottom). The peeling angle determines the asymmetric degree of plastic strain, and the deviation angle indicates the strain orientation. Accordingly, the peeling process can be divided into two categories: *δ* = 0°and *δ* ≠ 0°. To understand how peeling induces the shape morphing of plastic films, we simulated the peeling of a plastic PI film belt (30 μm thick, 5 mm wide) from an adhesive Kapton Tape on a rigid surface using finite-element analysis (FEA). When the film is peeled from one end at *ϕ* = 90° and *δ* = 0° (Supplementary Fig. 1), the film was bent at the detaching region and the two sides of the film experience different strains (Fig. 1c, left). The adhered side stretches while the other side contracts in the direction perpendicular to the detaching line due to the bending moment induced by the peeling force and adhesion force^27,28^. Because of the relatively small yield strain (Supplementary Figs. 2, 3), plastic deformation remains in the peeled film after peeling, and deformation differences between both sides of the film lead to its curling (Fig. 1c, right and Supplementary Movies 1–2). When peeling the PI belt at *δ* = 45° and *ϕ* = 180° (Fig. 1d and Supplementary Fig. 1b), the maximum principal plastic strain in the peeled film is at 45° toward the long axis. This causes the 2D films to morph into helices after peeling (Fig. 1d, right and Supplementary Movies 1–2), which resembles how a seedpod opens up^29^. By simply adjusting the peeling parameters, the magnitude and orientation of local strain can be controlled precisely and the 2D precursors can be morphed into complex 3D structures. For example, an orchid-like architecture^16^ with configurations of bending, folding, and helix was transformed from its 2D precursor film by peeling (Fig. 1e). Taken together, these results demonstrate that plastic peeling is a feasible method for shape morphing of plastic materials.

The plastic deformation for the shape morphing of plastic films during peeling was mainly attributed to the bending caused by the peeling force and adhesion force^27^. The bending process is affected by peeling parameters (e.g., peeling angle), geometry (e.g., thickness) and material properties (e.g., Young’s modulus and plastic yield strain) of the film and adhesive layer. Based on the equilibrium conditions during steady-state peeling (Supplementary Figs. 4–6), we developed a theoretical peeling model to quantitatively describe peeling-induced curling at zero deviation angle (Methods). In this case, the curvature (*k*_r_) of the film after release can be expressed as

1 $${k}_{\rm {r}}h=\left\{\begin{array}{c}{\varepsilon }_{0}\left(1-\bar{H}\right)\left(1/{r}^{2}-3+2r\right),\,1 \, < \, r\,\le\, 2,\\ {\varepsilon }_{0}\left(1-\bar{H}\right)\left(-7/{r}^{2}+3\right),\,r \, > \, 2\end{array}\right.$$

where $r={\varepsilon }_{B}/{\varepsilon }_{0}$, ${\varepsilon }_{0}$ denoting the plastic yield strain of the film and *ε*_*B*_ the maximum principal strain in the film during peeling; $\bar{H}=H/E$, *E* and *H* denoting the Young’s modulus and hardening modulus of the film, respectively; *h* is the film thickness. Note that *ε*_*B*_ is to be determined from system parameters through Eqs. 4–7 (Methods). The predicted curvature of the thin film after peeling, as well as the peeling force (per unit width), has been validated with numerical simulations based on finite element analysis (FEA) (Supplementary Fig. 7).

### The effect of peeling parameters to the curvature and chirality of the peeled films

**Fig. 2:**
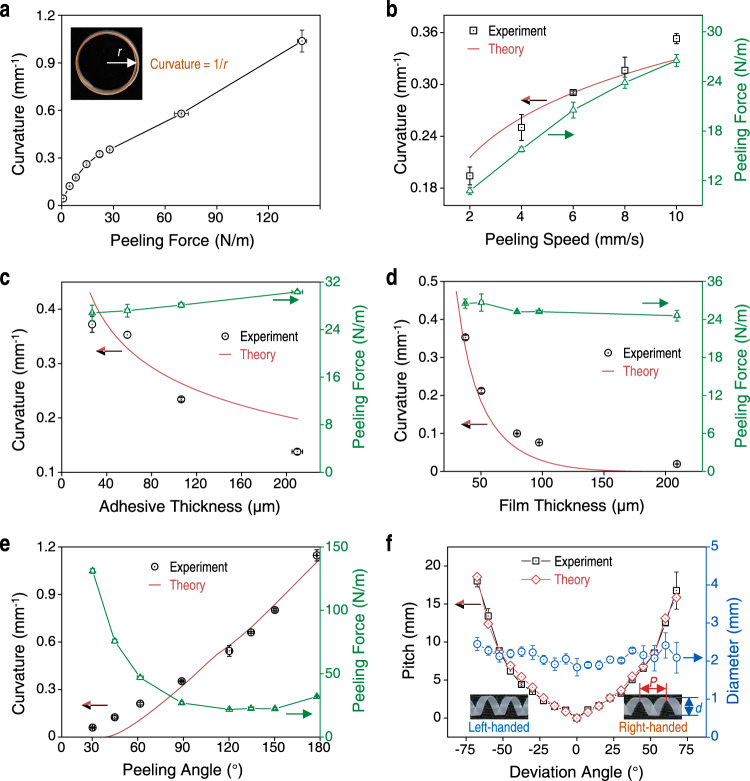
Effect of the adhesive layer and peeling parameters on shape morphing of plastic films. **a** Films peeled with higher peeling forces exhibited larger curvatures. Inset: optical photograph of a peeled PTFE film (37 μm thick, peeling angle of 90° and peeling speed of 10 mm/s). Curvature is defined as 1/*r*, where *r* is the radius of the circle. **b** Graph showing films that were peeled faster (measured as speed in mm/s) required larger peeling forces (green curve) and they resulted in more curly films (black dots and red curve). The theoretical results are quantitatively consistent with the experimental ones. **c** Graph of curvature and peeling force versus thickness of the adhesive layer (30–200 μm examined) showing films on thicker adhesives required slightly higher peeling forces to detach but they formed less curly shapes (lower curvature). **d** Graph showing plastic film thickness did not affect peeling forces (green curve) but thicker films displayed lower curvatures after peeling (black dots and red curve). **e** Curvature of the peeled film (black dots and red curve) increased as peeling angle increased from 30° to 180° and the peeling force (green curve) has a minimum at about 130°. **f** Graph of the pitch (black curve and red curve) and diameter (blue curve) of peeled films versus the deviation angle showing a positive deviation angle leads to right-handed helices, while a negative deviation angle results in left-handed helices. Larger deviation angles lead to helices with larger pitches. Insets: representative photographs of left- and right-handed helices. The pitch, *P*, is calculated by *P* = π*d* tan *δ*. The data in a-f are presented as mean ± s.d. of *n* ≧ 3 independent measurements. Source data are provided as a Source Data file.

We use PTFE as an example plastic film and cross-linked polydimethylsiloxane (PDMS) or commercial adhesive tapes as the adhesive layer to investigate the effects of related parameters on the shape morphing of the film theoretically and experimentally. The adhesion energy directly affects the peeling force required to detach the film and the bending degree of the plastic film near the detachment front. To investigate the effects of adhesion energy on peeling force and the bending degree of the peeled films, we fabricated adhesive layers with different adhesion energy using PDMS with different weight ratios of silicone base to crosslinking agent (from 10:1 to 60:1). Lowly cross-linked PDMS has more free and dangling polymer chains for the interfacial interaction with plastic films, and thus shows higher adhesion energy^30^. Higher adhesion energy led to greater bending of the film near the detachment front during peeling, which contributes to a larger plastic deformation of the film (Supplementary Fig. 8a). Thus, films peeled from adhesives with high adhesion energy required large peeling forces and were curled to large curvature (Fig. 2a, Supplementary Figs. 7a, 8b).

Due to the viscoelasticity of the adhesive layer, the fracture energy of the adhesive is positively associated with the peeling speed (Eq. S7)^30^. Higher peeling speeds required larger peeling forces (Fig. 2b), which led to larger deformation and greater bending of the plastic film near the detachment front during peeling (Supplementary Fig. 9). Thus, higher peeling speeds lead to larger curvature of the peeled film and the calculated results exhibit quantitative agreement with experimental results (Fig. 2b). Compared with the plastic film, the adhesive layer has a lower modulus and is usually stretched before the film and adhesive are separated (Supplementary Fig. 10). Thicker adhesive layers have also been shown to dissipate more energy within the bulk of the adhesive, making it harder to detach the film^31^. Thus, plastic films on thicker adhesives (between 30 and 200 μm) required greater peeling forces to detach (from 26.8 ± 1.3 N/m to 30.3 ± 0.3 N/m). However, the film peeled from thicker adhesives were less curly (curvature decreased from 0.37 to 0.13 mm^−1^), similar to the calculated results (Fig. 2c and Supplementary Fig. 10b), because a larger separation between the film and substrate was allowed and a larger area of adhesive was involved in the detaching process, leading to lower bending curvature (Supplementary Fig. 10a), and smaller plastic deformation in the film.

Besides the adhesive layer, the thickness and Young’s modulus of the plastic films can also affect the bending during peeling and therefore the curvature of the peeled films. Thicker plastic films required more force to bend, while the force required to peel thick films from the adhesive remained largely unchanged (Fig. 2d and Supplementary Fig. 7b). Thus, thicker films were less curly than thinner films due to the difference in bending stiffness (Supplementary Fig. 11a). Therefore, thicker films result in peeled films with smaller curvatures as confirmed by both experimental results and calculated results (Fig. 2d and Supplementary Fig. 11b). This was also experimentally observed for other plastic films such as PI and PET (Supplementary Fig. 12). Furthermore, the Young’s modulus of the films exhibits a similar effect on the peeling process, that is, films with higher modulus bent less during peeling and showed lower curvatures after peeling due to larger bending stiffness (Supplementary Figs. 7c, 13).

As a vital parameter, the peeling angle exhibited significant effects on the peeling process. With peeling angles increasing from 30° to 180°, the bending degree of the film at the detached region gradually increased (Supplementary Fig. 14a), leading to larger plastic deformation and increased curvature in the peeled films (Supplementary Fig. 14b). The predicted curvatures at different peeling angles from our peeling model are consistent with and further confirmed the experimental results quantitatively (Fig. 2e). FEA results of plastic PI films peeled at different peeling angles also verified that the curvature increases with the peeling angle, in agreement with the experimental results (Supplementary Fig. 15). At *δ* = 0°, tube-like structures formed after peeling because the film was stretched or compressed in the direction perpendicular to the detaching line and along the longitudinal axis of the film. Interestingly, previous studies have shown that expansions of different parts in seed pods^29^ and hydrogel sheets^32^ oriented at an angle (other than 0° and 90°) to the longitudinal axis will lead to helical structures. To regulate the direction of plastic strain, we adjusted the deformation direction of the film by changing the directions of peeling force and detaching line, that is the deviation angle, *δ* (Supplementary Fig. 16a). At different deviation angles, the peeled films formed cylindrical helices with different chirality and pitches (Fig. 2f and Supplementary Fig. 16b). Positive deviation angles resulted in right-handed helices while negative angles formed left-handed helices. Pitch (*P*) of the helical structures can be calculated as

2 $$P=\pi d\,\,{{\tan }}\,\delta$$

where *d* is diameter of the helical film (methods and Supplementary Fig. 16a). Our experimental data were consistent with the calculated *P* values (Fig. 2f). Through simple manipulation of deviation angles, similar chiral cylindrical helices were also obtained with other plastic films such as PI and PE (Supplementary Fig. 16c, d).

### Complex 3D structures by regulating the peeling process

**Fig. 3:**
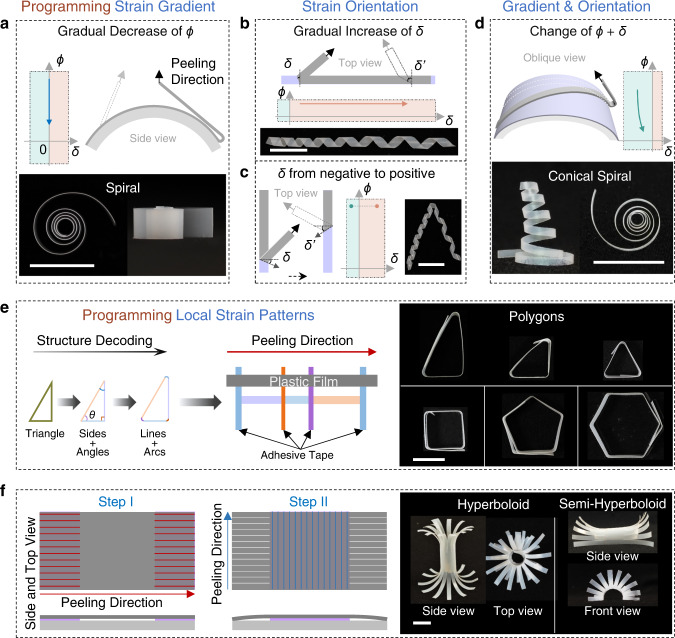
Peeling plastic films with pre-set parameters and methods results in complex structures. **a** Plastic films peeled from an arcuate surface with a gradually changing peeling angle (*ϕ*) result in spiral-like structures. Top: schematic showing the peeling process. Bottom: top view (left) and side view (right) photographs of the spiral-like structure. **b** Plastic films peeled with gradually increasing deviation angle (*δ*) form cylindrical helices with gradually increasing pitch. Top: schematics showing the peeling process with *ϕ* of ~180°. Bottom: photographs of the helices. **c** Plastic films peeled with *δ* changing from negative to positive (left schematic) form cylindrical helices with different chirality (right photograph). Schematics show top views of the peeling process at *ϕ* of 180°. In **a**–**c** schematics, the dotted outlines and gray arrows indicate the final state of the peeling process. **d** Films peeled with simultaneously changing *ϕ* and *δ* form conical spirals. Top: oblique view schematic of the peeling process, where the plastic film (gray) adhered at an angle to the generatrix of the arcuate substrate. Bottom: side view (left) and top view (right) photographs of the conical spiral films. In **a**–**d**, the *ϕ*-*δ* phase diagrams indicate the change of *ϕ* and *δ* during the peeling process. **e** Left schematic shows the structure decoding of polygons with a right triangle as an example that the sides and interior angles can be viewed as lines and arcs which correspond to the intervals and widths of the adhesive tapes. Right schematic (top view) shows the arrangement of adhesives (orange, purple and blue strips) for the triangle and peeling direction (red arrow) with *ϕ* of 180°. Right photographs: triangles, quadrangle, pentagon and hexagon. **f** Two-step peeling with orthogonal peeling directions results in hyperboloids. Left: top view (top) and side view (bottom) schematics of the two-step peeling process. Parallel red and blue lines indicate the adhesion regions on different sides of the plastic film. The line direction indicates the peeling direction. The white lines mean that these parts were cut into parallel strips. Right: photographs of the hyperboloids. All the scale bars are 10 mm.

By gradually changing one or more of these peeling parameters during peeling, we can predict and realize a variety of complex 3D shapes. For example, when we decreased the peeling angle gradually by peeling the plastic film from an arcuate surface, the curvature of the peeled film was gradually decreased, and spiral-like structures were obtained (Fig. 3a and Supplementary Movie 3). Gradually increasing the deviation angles resulted in cylindrical helices with gradually increasing pitch (Fig. 3b and Supplementary Movie 4). If deviation angles change between negative and positive during peeling, helices with different chirality can be acquired in the same plastic film (Fig. 3c and Supplementary Movie 5). When the peeling and deviation angles were changed simultaneously, both curvature and pitch of the peeled film changed, and the films were transformed into conical spiral shapes (Fig. 3d and Supplementary Movie 6). As indicated in the phase-like diagram of the peeling angle (0°<| *ϕ* |≤ 180°, where a negative angle means adhesion on the other side of the film) and deviation angle (0° ≤ |*δ*| < 90°), the bending direction and chirality can be switched by changing the peeling and deviation angles between positive and negative values, respectively (Supplementary Fig. 17a). In general, the final 3D structures of the peeled film can be predicted and fabricated by adjusting the peeling angle and deviation angle (Supplementary Fig. 17b).

**Fig. 4:**
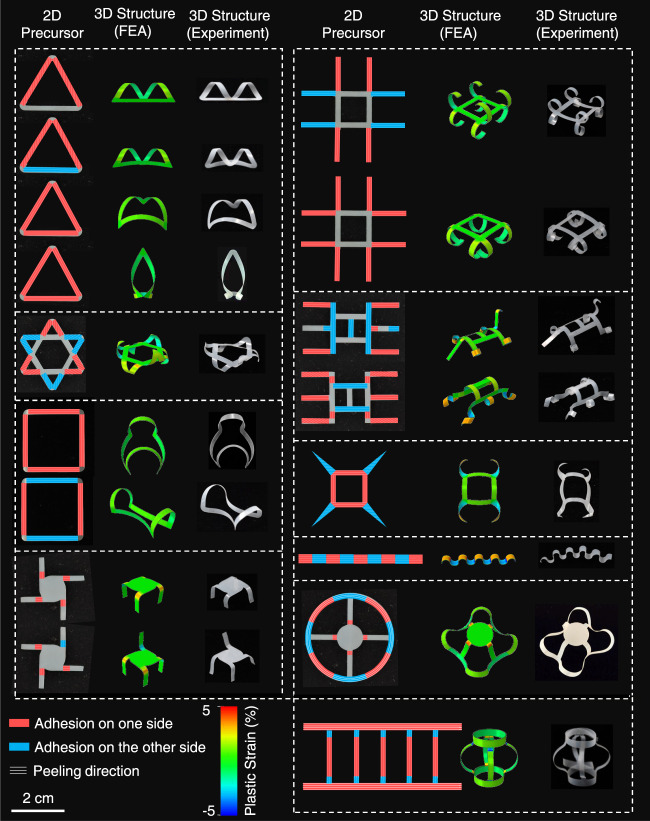
Complex 3D geometries programmed by designing the layout of the 2D precursors and regulating the peeling process. Multi-step peeling morphs the 2D precursors of PTFE film into complex 3D geometries by regulating the adhered parts and peeling parameters, including the peeling angle and the deviation angle. Red and blue parts indicate the adhered regions on different sides of the plastic film and the direction of the white lines indicates the peeling direction. In all cases, the color in the FEA results reflects the maximum principal plastic strain.

Through discrete peeling of the film belt, local curvature can be programmed and thus different polygonal shapes such as triangle, quadrangle, pentagon and hexagon were also obtained by simply arranging the adhesive layers with predetermined widths and intervals according to the interior angles and side lengths of the targeted shape and peeling the film under the according parameters (Fig. 3e and Supplementary Movie 7). Taking right triangle with an interior angle of 60° and shortest side of 10 mm and the 80 μm thick PTFE film at a peeling angle of 180° as an example, the diameter of the peeled film, *d* is ~2 mm and thus the widths of adhesive tapes (*w*_*a*_ = (180 - *θ*)π*d*/360, *θ* is the interior angle) are 2.62 mm, 1.57 mm and 2.09 mm for interior angles of 30°, 90°, and 60°, respectively and intervals are 17.3, 10 and 20 mm, respectively (Fig. 3e, left). When peeling the film at peeling angle of 180°, the default right triangle was obtained (Supplementary Movie 7). Furthermore, complex 3D structures can be designed and fabricated through a multistep peeling process. For example, a two-step peeling process with orthogonal peeling directions and opposite curling directions can form hyperboloids (Fig. 3f and Supplementary Movie 8). In step I, a PTFE film (4 cm × 2 cm) was attached on two separated adhesive tapes (1 cm × 2 cm with an interval of 2 cm) and peeled off along the red line (Fig. 3f, left); in step II, the other side of the PTFE film was adhered on the adhesive tape (2 cm × 2 cm) and peeled off along the blue line with different peeling angles for the hyperboloids and semi-hyperboloids, before which the parts adhered in the first step were cut into parallel strips along their curling direction. More complex final 3D geometries can also be programmed by designing the layout of the 2D precursors and peeling them along the default routes (adhered regions, peeling direction, and peeling angle). Using this method, we obtained seventeen highly complex 3D geometries from ten 2D precursors and remarkably good agreement between experimental results and FEA predictions further verified the versatility of this shape-morphing strategy and the accuracy of the models (Fig. 4). Apart from the polymer films, the peeling-induced shape morphing strategy is also applicable to other materials capable of plastic deformation, such as metals (copper, silver, aluminum, nickel, titanium, and iron), semiconductor polymer films (poly(3-hexylthiophene-2,5-diyl), P3HT), filter paper, and composite films ranging from centimeter to micrometer (Supplementary Fig. 18).

### Peeling-induced shape morphing for 3D devices

**Fig. 5:**
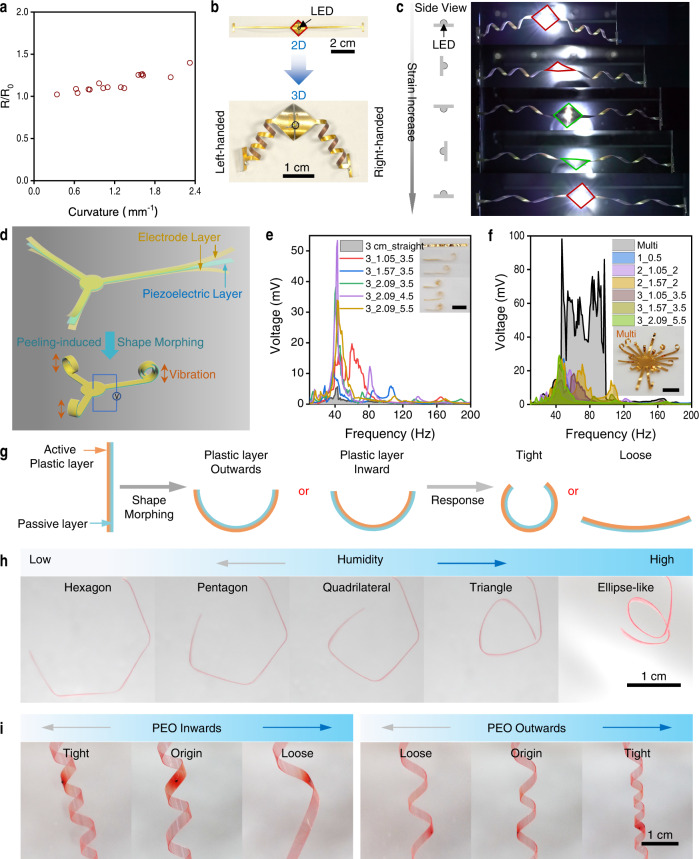
Peeling-induced shape morphing for 3D and 4D electronics. **a** Graph showing that increased bending (measured as curvature) results in only a slight increase in resistance for Au-PTFE films. **b** A 3D circuit morphed from the 2D one with a light emitting diode (LED) connected to two helical wires with different chirality. **c** The stretching processes of the circuits in (**b**) with the LED rotating during the stretch but without compromising the light intensity. Left: schematic showing the position changes of the LED. **d** Schematic illustrations of the 3D cantilever-based piezoelectric systems. The piezoelectric system is of sandwich structure with two electrode layers and a piezoelectric layer. The 2D cantilevers can be transformed into 3D spiral-like structures by peeling-induced shape morphing. **e**, **f** The generated voltages of the single cantilever (**e**) and multi-arm cantilever (**f**) piezoelectric systems at different frequencies indicating the peeling-induced 3D cantilevers can sense broader vibrational frequencies and generate higher voltage than the straight one and the integration of the 3D cantilevers can further enhance the performance of the piezoelectric system. Scale bars are 1 cm. **g** Schematic illustrating the shape morphing of a bilayer actuator composed of an active plastic layer (PEO in our case) and a passive layer (PDMS). Subsequent exposure to humidity transforms the bilayer into 4D shapes. **h** Photographs showing polygonal PEO/PDMS films with PEO outwards transformed from hexagon to triangle and ellipse-like shape as humidity increases. **i** Humidity-responsive transformation of PEO/PDMS cylindrical helices. With active PEO inwards, the cylindrical helix became tight under lower humidity and loose under higher humidity. On the contrary, the cylindrical helix with PEO outwards exhibited opposite humidity-responsive motions. Source data are provided as a Source Data file.

As a proof-of-concept, we used the peeling-induced shape morphing method to fabricate 3D electronics. Before that, we investigated the effect of the metal layer on the shape morphing of the polymer substrate (Supplementary Fig. 19a–c). With the bilayer of PTFE (557 MPa, 90 μm) and copper (25–1000 nm by thermal evaporation) as an example, the curvature of peeled PTFE-Cu films almost was unchanged with Cu thickness <500 nm and increased slightly with Cu thickness of 1000 nm. These results indicate that the deposited metal (~100 nm) for electronics will almost not affect the shape morphing of plastic films like PET, PI, and PTFE (tens of microns thick). Further, the effect of shape morphing on the conductivity was investigated. With gold (Au, 70 nm) deposited on the surface of PI (14 μm), PET (19 μm), and PTFE (37 μm) plastic films (named Au-polymer film), these films can be morphed into various structures and the resistance was almost unchanged with the curvature <0.6 mm^−1^ and slightly increased (<40%) even under rather large curvatures (<2.3 mm^−1^ for Au-PTFE, <1.5 mm^−1^ for Au-PET, and <0.8 mm^−1^ for Au-PI) caused by the stretch of the film (Fig. 5a and Supplementary Fig. 19). For the electronic devices, the final structures and curvature of the Au-polymer films could be precisely regulated by our peeling-induced shape morphing strategy to satisfy the requirements for practical applications and the resistance changes caused by shape morphing could be predicted and taken into account in the circuit design. Further, the Au-PTFE films morphed into cylindrical helices could also be stretched to more than 9 times their length without significant change in resistance (<2%, Supplementary Fig. 19l, m). With the peeling-induced shape morphing strategy, a 3D orchid-like circuit was fabricated from the 2D ones without degradation of the performance (Supplementary Fig. 19n and Supplementary Movie 9), and the 3D circuits fabricated from the non-stretchable 2D circuits exhibited good stretchability with the light intensity almost unchanged during the stretch (Fig. 5b, c and Supplementary Fig. 20) indicating that peeling-induced shape morphing is a promising and simple strategy for constructing 3D circuits. These 3D structures also endowed the circuits with expanded functions. For example, the lamp with the wires of opposite chirality rotated a full circle under 50% strain which provides a new clue for strain sensing by measuring the rotation angle (Fig. 5b, c). The 3D circuits also could provide accommodation for sensors in the 3D space endowing the devices with good spatial resolution (Supplementary Fig. 21).

Piezoelectric devices have shown advantages in mechanical sensors and energy harvesters and structural designs also facilitated the progress in these systems^5^. With our peeling-induced shape morphing strategy, the commonly used planar plastic piezoelectric polymer systems could also be easily transformed into 3D shapes, enhancing and extending their performance and functions (Fig. 5d–f and Supplementary Fig. 22). Here, a piezoelectric system of poly(vinylidene fluoride-co-trifluoroethylene) (P(VDF-TrFE) with thicknesses of ~14 μm) as piezoelectric layer and layers of metals (Cr/Au, 5 nm/70 nm in thickness) as electrodes on the top and bottom surfaces was taken as an example (Fig. 5d). The 2D piezoelectric polymer films were transformed into spiral-like structures by peeling the film from an arcuate surface with PDMS (60:1) as adhesive layers (Supplementary Fig. 22a) and the exquisite structure of these spirals were regulated by the lengths of the adhered part and link belt (Supplementary Fig. 22b, the structures are named as Length of Piezoelectric Film_Length of Adhered Part_Length of Link Belt). With the piezoelectric film as cantilevers, external vibrations can excite the piezoelectric system and thereby produce electrical power that can be sensed or harvested through the two metal electrodes (Supplementary Fig. 22e). The 3D cantilever can sense broader vibrational frequencies (24–92 Hz with voltages > 1 mV for 3_1.05_3.5) and generate higher voltage (peak voltage 53.4 mV for 3_2.09_4.5) than the 2D precursor film (1.5 mm × 3 cm, 27–53 Hz with voltage > 1 mV, peak voltage 5.7 mV) (Fig. 5e and Supplementary Fig. 22 g). By integrating different 3D cantilevers into a multi-arm cantilever piezoelectric system, the performance of variation sensing and energy harvesting were further enhanced (Fig. 5f, 19–184 Hz with voltage > 1 mV and maximum voltage of 98.2 mV). These results indicate the potential advantages of our peeling-induced shape-morphing strategy in the fabrication of 3D devices.

This shape morphing strategy is also applicable to other complex films, such as bilayer films of plastic film and elastomer although the elastomer layer will decrease the curvature of the peeled films (Supplementary Fig. 23). These results indicated the peeling-induced shape morphing strategy can also be further used to diversify the shapes of actuators^33^. With a bilayer film of polyethylene oxide (PEO)/PDMS as an example, the film can bend with the humidity change working as a hygroscopic actuator, where the PEO layer is plastic and easy to expand at high humidity. With the peeling-induced shape morphing strategy, the PEO/PDMS film could be transformed into a variety of structures as the initial shapes, such as polygons and cylindrical helices by virtue of the plasticity of the PEO layer (Supplementary Fig. 24a–d). The morphed 3D structures can further transform into other shapes under changing humidity, (called 4D shape transformation, Fig. 5g–i). For example, a morphed PEO/PDMS triangle progressively transformed into a quadrilateral, pentagon and hexagon when moved from an indoor environment (~60% RH at 25 °C) to a dry tank containing anhydrous calcium chloride (<10% RH), but formed an ellipse-like structure when exposed to wet gas (Fig. 5h and Supplementary Movie 10). By switching locations of the PEO and PDMS layers during peeling, similar cylindrical helices exhibited opposite transformation behavior under the same stimulation. Cylindrical helices of PEO/PDMS films with an inward PEO layer tightened and formed more spirals under low humidity and loosened at high humidity, whereas films with an outward PEO layer displayed the opposite behavior (Fig. 5i and Supplementary Movie 11). These results demonstrate that together with stimuli-responsive materials, peeling-induced shape morphing can be used to program and diversify the shapes of actuator films. The circuits on the PEO/PDMS film can be transformed to 3D shapes and keep working during humidity-induced shape transformation, indicating the potential application in 4D electronics (Supplementary Fig. 24e, f and Supplementary Movie 12).

This peeling-induced shape morphing strategy can also be extended to fabricate 3D elastomer films from their precursor solutions by using a plastic film as the template based on our peeling-induced shape morphing strategy (Supplementary Fig. 25). As a proof-of-concept, 3D-shaped PDMS films were fabricated by spreading the precursor solution of PDMS on a PTFE film as the template and allowing the solution to partially cure (Supplementary Fig. 25a). Then the bilayer of PTFE and semi-cured PDMS was transformed into an expected 3D structure using peeling-induced shape morphing. After that, the PDMS was further cured and a 3D PDMS film was obtained after removing the PTFE template (Supplementary Fig. 25c). Any functional tattoos on the plastic film (for example, the patterned gold circuit in our case) can also be transferred to the 3D elastomer film (Supplementary Fig. 25b, d), making it possible to form a fully functional 3D stretchable electronics.

## Discussion

In summary, we have demonstrated using peeling-induced asymmetric plastic deformation as a simple and versatile strategy to program the 3D structures of inert plastic materials from 2D precursors with dimensions ranging from millimeters to micrometers. Our strategy applies to most materials capable of plastic deformation and the final geometries could be predicted and programmed by designing the 2D precursor structures and modulating the peeling parameters. The introduction of active materials endows the peel-induced 3D plastic films with additional capabilities to transform their shapes further. In addition, using the plastic films as templates, 3D structures of elastomer films could also be programmed. Proof-of-concept demonstrations of 3D circuits with plastic films as substrates and 3D piezoelectric systems by peeling-induced shape morphing indicate that this strategy can potentially be used for manufacturing a vast variety of 3D flexible electronic devices. Complementary to other strategies causing plastic strain such as mechanical loading apparatus^17^, focused ion beam^34,35^, and folding origami^36,37^ (Supplementary Table S2), our peeling-induced shape morphing strategy is expected to further bring prosperity to the 3D construction of plastic materials.

## Methods

### Finite element analysis (FEA) of the peeling model

Full three-dimensional (3D) finite element analysis (FEA) for simulating the detaching and releasing behavior of PI film was carried out with commercial software ABAQUS. Two steps analyses were adopted. The polyimide, fully attached to a substrate through an adhesive layer, is subsequently detached from one end (first step), and then released to form the curved (or helical) structure (second step). A zero thickness cohesive element model using the quadratic stress criterion was utilized to predict delamination initiation of the adhesive layer. The mixed-mode linear energy-based damage propagation model based on Benzeggagh-Kenane (BK) law was used to characterize the damage evolution. Elastic-plastic model was used to capture the mechanical behavior of the PI film. Young’s modulus (*E*) and Poisson’s ratio (*ν*) are *E* = 2.5 GPa and *ν* = 0.35 for PI. The yield stress and corresponding plastic strain were derived from the measured data (Supplementary Fig. 2). The tensile (represented with subscript n) and shear (represented with subscript s) stiffness (*E*_n_ and *E*_s_), initial damage stress (*t*), and fracture energy (*G*) of the adhesive layer are *E*_n_ = 52 MPa mm^−1^, *E*_s_ = 16.5 MPa mm^−1^, *t*_n_ = 0.52 MPa, *t*_s_ = 0.55 MPa, *G*_n_ = 5.2 N m^−1^, *G*_s_ = 14.67 N m^−1^, respectively. Eight-noded 3D solid elements and eight-noded 3D cohesive elements were used for the polyimide and adhesive layer, respectively, and refined meshes were adopted to ensure the accuracy.

Some 3D structures in Fig. 4 can be simulated directly using the two-step peeling-releasing method. These structures can be obtained through one peeling process (e.g., the structure in Row 1 Column 1) or multiple peeling processes that do not influence each other (e.g., the structures in Row 5 Column 1 and Row 1 Column 2), followed by the releasing process. However, this method suffers from sophisticated boundary conditions and convergence problems for some structures (e.g., the structure in Row 3 Column 1). To overcome these problems, we developed a general simulation strategy that can be applied to complex peeling processes. First, we simulate each adhesive part of a 2D precursor using the two-step method individually. Second, the deformation of each relaxed structure is applied to the corresponding part of the film in undeformed state, yet with the plastic part of the material constitutive law removed. Since the film only undergoes elastic deformation in the relaxation stage (the ef part in Supplementary Fig. 5b), the residual plastic strain/stress in the film can be equivalently calculated as an eigenstrain/eigenstress in the undeformed film. Finally, we apply the eigenstrain/eigenstress fields extracted from each structure in the second step to the corresponding part in the 2D precursor to obtain the relaxed 3D configuration. In the final step, the 2D precursor film is also purely elastic.

In addition, two-dimensional (2D) plane-stress finite element simulations were implemented to validate our theoretical model with zero deviation angle. The constitutive laws of materials and the traction-separation law for adhesive layer adopted in 2D simulations are the same as those in the 3D cases.

### Governing equations for shape morphing of plastic film with zero deviation angle

To better understand the mechanism of the peeling-induced shape morphing phenomenon and predict the curvature of the released film after peeling, we developed a mechanical model for the peeling and release processes of a thin film. The film material was assumed to be elastoplastic with linear kinematic hardening, a common constitutive relation for polymeric and metallic materials (Supplementary Fig. 4).

The model results in a system of closed form governing equations considering geometric and material parameters of the film and adhesive layer as well as peeling parameters. Introduce the dimensionless variables:

3 $$r=\frac{{\varepsilon }_{B}}{{\varepsilon }_{0}},\,\,\bar{P}=\frac{P_0}{{Eh}},\,\,{\bar{\varGamma }}^{a}=\frac{{\varGamma }^{a}}{{Eh}},\,\,\bar{H}=\frac{H}{E},\,\,{\bar{M}}_{Q}=\frac{{M}_{Q}}{{Ew}{h}^{2}},\,\,{\bar{E}}^{a}=\frac{{E}^{a}h}{{Et}}$$

where *E*, *H*, *ε*_0_ and *h* denote the elastic modulus, hardening modulus, yield strain and thickness of the plastic film, respectively; *E*^*a*^, *Γ*^*a*^ and *t* denote the elastic modulus, effective adhesion energy and thickness of the viscoelastic adhesive, respectively. In Eq. (3), the maximum principal strain in the film *ε*_*B*_, the bending moment *M*_Q_ of the film at detachment front and the peeling force *P*_0_ are to be determined. With the dimensionless variables in Eq. (3), the governing equations can be written as

4 $$\left(1-{{\cos }}\,\,\phi \right)\bar{P}-{\bar{\varGamma }}^{a}=\left\{\begin{array}{cc}\frac{\left(1-\bar{H}\right){\varepsilon }_{0}^{2}}{6}\left(\frac{2}{r}+1\right){\left(1-r\right)}^{2},& 1 < r\le 2,\\ \frac{\left(1-\bar{H}\right){\varepsilon }_{0}^{2}}{6}\left(\frac{10}{r}-15+6r\right),& r > 2,\end{array}\right.$$

5 $$\bar{P}\left[1-{{\cos }}\left(\phi -{\phi }_{0}\right)\right]-6{\bar{M}}_{Q}^{2}=0,$$

6 $${\varepsilon }_{0}r-6{\bar{M}}_{Q}=0,$$

7 $$144{\bar{M}}_{Q}^{2}-\sqrt{3{\bar{E}}^{a}}{{{\tan }}}^{2}\,\,{\phi }_{0}=0$$

where *ϕ*_0_ denotes the inclining angle of the film at detachment front. The curvature of the film after peeling and release can be calculated by using Eq. (1) after *r* is solved from Eqs. (4)–(7).

### Numerical validation of the theoretical model

To validate the theoretical model, 2D finite element simulations were conducted. All the parameters used in the theory and simulations have been summarized in Supplementary Table S1. Some typical values of the parameters adopted for validation examples are also shown. Note that these values may change depending on the quantity of interest in different groups of simulations. Due to equation (S7), the viscoelastic effect of the adhesive is studied directly via *Γ*^a^ and the values of associated parameters *v,κ,n,a*_*T*_ and ${\varGamma }_{0}^{{{{{{\rm{a}}}}}}}$ are not specified in Supplementary Table S1.

Supplementary Fig. 7 shows the comparison of FEA and modeling results with different parameters as the control variable. In all cases, the predictions from our theoretical model are consistent with the FEA results. Some quantitative errors can be ascribed to the simplifications applied on the constitutive law of the film in theoretical analyses. The accuracy of the modeling predictions can be expected to improve by relaxing these simplifications, which will be explored in the future.

### Plastic film and characterization of the plastic deformation

The plastic films used in this work include polyethylene terephthalate (PET), polyimide (PI), polytetrafluoroethylene (PTFE), polyvinylpyrrolidone (PVP, Sigma-Aldrich/PVP40), polyvinyl butyral (PVB, Sigma/P110010), polyethylene oxide (PEO, Mv = 1,000,000, Sigma-Aldrich), polyvinylidene fluoride (PVDF), poly(vinylidene fluoride-co-hexafluoropropylene) (PVDF-HFP), polylactic acid (PLA), polyethersulfone (PES) filter membrane, weighing paper, aluminum (Al) foil and copper (Cu) foil. Among them, PI, PET, PTFE, PES filter membrane, weighing paper, Al foil and Cu foil are commercial films with different thicknesses. The PVP, PVB, PEO, PVDF, and PVDF-HFP films were fabricated from their solution (4 wt%, PVP and PEO in water; PVB in isopropyl alcohol, PVDF and PVDF-HFP in *N*-Methyl-2-pyrrolidone) through solvent volatilization. The PLA belt was fabricated via hot drawing with PLA at 250 °C as the sample for shape morphing at the microscale.

The stress-strain curves of these films were obtained by a mechanical tester (C42, MTS Systems Corporation) at 30 mm/min (Supplementary Fig. 2). Stress-strain hysteresis loops of plastic films were conducted at different strains (0.001, 0.0025, 0.005, 0.0075, 0.01, 0.015, 0.02, 0.03, 0.04, 0.06, 0.08, and 0.10) to get the plastic strain-strain curves (Supplementary Fig. 3).

### Fabrication of adhesive layer

The adhesive layers were fabricated with polydimethylsiloxane (PDMS) (Sylgard 184, Dow Corning, United States) consisting of a silicone base and a crosslinking agent^30^. The PDMS with different ratios of the silicone base and crosslinking agent (10:1, 20:1, 30:1, 40:1, 50:1 and 60:1) were mechanically stirred and degassed by centrifugation at 2907 × *g* for 5 min. Then, the precursors were spread on a plasma-treated PET film (100 μm) using the blade coating technique at different thicknesses (~25, 50, 100 and 200 μm determined). After that, the precursors are cured at 80 °C for 2 h and adhesive layers with different interface fracture energy were obtained. Some commercial tapes (scotch tape and Kapton tape) were used as the adhesive layer directly.

### Peeling-induced shape morphing

The peeling process was conducted using a tensile machine and home-made slidable substrate to keep constant peeling angles. The adhesive layer was fastened on the substrate with double-sided tape, and then the plastic films (width 4–5 mm) adhered on the adhesives for the subsequent controllable peeling. Peeling was conducted with one end of plastic film on the tensile machine. The peeling speed (the movement speed of the detaching line) of the plastic films was fixed at 10 mm/s by adjusting the pulling speed of the tensile machine and the movement of the substrate. During the peeling process, the dynamic three-phase (plastic film, adhesive layer and air) line was recorded with a camera (SONY).

The peeling process involved several parameters. The curvature of the peeled-off films is related to the adhesion force, peeling speed, adhesive thickness, plastic film thickness, plastic film modulus and peeling angles. The pitch of cylindrical helices can be adjusted with the deviation angle. The peeling-induced shape morphing also could be programmed by adjusting single or multiple parameters during peeling. Here, the fabrication of several structures is demonstrated.

#### Spiral

The PTFE film (thickness of 80 μm, width of 5 mm and length of 10 cm) adhered on the flank of a cylinder with a diameter of 9 cm with Kapton tape as the adhesive layer (Fig. 3a). The PTFE film was vertical to the cylindrical generatrix. The peeling process started from one end of the PTFE film with a vertical pulling direction. During this process, the peeling angle decreased gradually with movement of the detaching line, leading to a gradually decreasing curvature after peeling.

#### Cylindrical helices with gradually increasing pitch

The PTFE film (thickness of 80 μm, width of 2 mm and length of 10 cm) was adhered horizontally on a vertical plate with Kapton tape as the adhesive layer and peeled at a peeling angle of 180° (Fig. 3b). When peeled from one end, the deviation angle increased with the proceeding of the contact line leading to the increasing pitch (Fig. 3b).

#### Tendril-like structure

The PTFE film (thickness of 80 μm, width of 2 mm and length of 10 cm) adhered vertically on the left side of a vertical plate with Kapton tape as the adhesive layer and peeling angle of 180° (Fig. 3c). The peeling process started from the bottom end of the PTFE film with a vertical pulling direction. During the peeling process, the vertical plate moved right resulting in the change of deviation angle from negative to positive leading to the cylindrical helices with different chirality (Fig. 3c).

#### Conical spiral shape

The PTFE film (thickness of 80 μm, width of 2 mm and length of 10 cm) adhered on the flank of a cylinder with diameter of 9 cm with Kapton tape as the adhesive layer (Fig. 3d). The PTFE film came to a certain angle to the cylindrical generatrix (~60°). The peeling process started from one end of the PTFE film. During this process, the peeling angle decreased gradually while the deviation angle increased gradually with the movement of the contact line resulting in a conical spiral shape after peeling (Fig. 3d).

#### Polygons

The adhesive layer was parallelly aligned with certain intervals. The width of the adhesive layer (*w*_*a*_) was calculated from the interior angles *ɵ* and the diameter of the peeled-off film, *d*, where *w*_*a*_ = (180−*ɵ*) π*d*/360. The intervals are equal to the corresponding side length. Taking right triangle with interior angles of 60° and the shortest side length of 10 mm as an example, the diameter of peeled-off PTFE film (thickness of 80 μm, width of 4 mm) with Kapton tape as the adhesive layer and a peeling angle of 180° is 2 mm; thus, the widths of adhesive layers should be 2.62, 1.57, 2.09 and 2.62 mm, respectively, and the corresponding intervals should be 17.3, 10 and 20 mm, respectively (Fig. 3e). The PTFE film (thickness of 80 μm, width of 4 mm) was adhered vertical to the adhesive layer and peeled off at peeling angle of 180°. For an isosceles right triangle with a short side length of 10 mm, the widths of adhesive layers should be 2.36, 1.57, 2.36, and 2.36 mm, respectively, and the corresponding intervals should be 10, 10, and 14.1 mm, respectively. For an equilateral triangle with a side length of 10 mm, the widths of adhesive layers should be 2.09, 2.09, 2.09, and 2.09 mm, respectively, and the corresponding intervals should be 10, 10 and 10 mm, respectively. For the equilateral polygon with a side length of 10 mm, the widths of adhesive layers are 1.57, 1.26, and 1.05 mm with intervals of 10 mm for quadrangle, pentagon and hexagon, respectively.

#### Hyperboloid

Hyperboloid structure was fabricated by a two-step peeling process. The PTFE film (thickness of 80 μm) was cut into 2 cm × 4 cm. At the first step, two parallel adhesive tapes were fixed on a plate with the tape length of 2 cm, width of 1 cm and an interval of 2 cm. The PTFE film was peeled off along the long side of the PTFE film. At the second step, the adhesive tape was 2 cm × 2 cm and the PTFE film was adhered with another side and peeled off along the short side of the PTFE film. Before that, the adhered parts in the first step were cut into parallel strips along the long side (Fig. 3f). The curvature of the two parts can be tuned separately with different peeling angles.

#### More complex 3D structures

Based on the obtained rules to regulate the shape morphing of plastic films, much more complex structures were fabricated with programmed 2D precursors and peeling processes. As shown in Fig. 4, the same 2D precursor can be transformed to different 3D structures by changing the adhesion side and peeling direction. For example, the same triangle frame can be turned into more than four different 3D structures. With different 2D precursors, various 3D structures were obtained which are inaccessible by other methods.

### Fabrication of circuit on the plastic film and resistance change measurement

The circuits were fabricated by physical vapor deposition. Prior to metal depositions, the plastic films were treated with oxygen plasma to increase the adhesion of the metal and polymer film. The treated films were pasted onto the trial of the thermal evaporation machine (Nano 36, Kurt J. Lesker). After vacuumed with a molecular pump for 2 h, metals (Au and Cu) were deposited on the plastic film. To investigate the effect of the deposited metal layer on the shape morphing of plastic films, Cu and PTFE (~90 μm) were selected and the thicknesses of the Cu layer included 25, 50, 100, 250, 500, and 1000 nm. Then, the bilayer films were cut into belts with width of 5 mm and peeled from the substrate with Kapton tape as the adhesive layer at the peeling angle of 90°.

For circuits, Au was deposited at the rate of 0.4 Å s^−1^. The final thickness of gold was 70 nm. For circuits with certain patterns, stainless steel masks were covered on the plastic film towards the gold source^38,39^. The Au-polymer films were cut in 5 mm × 50 mm. The resistance changes with the strain were measured with a semiconductor parameter analyzer (Keithley 4200-SCS, Tektronix) under gradual extension by a mechanical tester (C42, MTS Systems Corporation) at 30 mm/min. The resistance changes at different curvature were obtained by measuring the resistance of the Au-polymer film with a width of 2 mm (thickness of PI, PET and PTFE: 14, 19 and 37 μm) before and after peeling-induced shape morphing.

For the functional 3D circuits, the 2D circuits were fabricated by depositing Au on the predesigned 2D precursors PTFE film (treated with O_2_ plasma before metal deposition) and commercial devices (LEDs and photoresistors) were connected to the circuits with anisotropic conductive film (ACF). After that, the 2D circuits were transformed into 3D ones with the peeling-induced shape morphing strategy (Kapton tape as the adhesive layer).

### Bilayer films of plastic film and elastomer

The peeling-induced shape morphing strategy also applies to the bilayer films of plastic layer and elastomer layer. Here, PTFE (~90 μm) and PDMS were used to investigate the effect of the thickness and modulus of the elastomer layer on the shape morphing of the bilayer film. The PDMS precursor was coated on the plasma-treated PTFE by blade-coating and the thickness was regulated from ~20 to ~400 μm. The modulus of the PDMS was regulated by changing the ratios of the silicone base and crosslinking agent. Then, the bilayer films were cut into belts with width of 5 mm and peeled from the substrate with Kapton tape as the adhesive layer at the peeling angle of 90°.

### Fabrication of responsive bilayer films and their humidity responsiveness

The bilayer film was composed of PEO and PDMS. First, PEO film was prepared with 9 mL 4 wt% PEO aqueous solution in a petri dish with the diameter of 9 cm through solvent volatilization in an oven at 60 °C. Then, PDMS precursor (silicone base: crosslinking agent = 10:1) stained in red with oil red was coated on the PEO film by spin coating (1000 rpm, 90 s). Then, the precursor was cured in an oven at 80 °C for 2 h and the PEO/PDMS bilayer films were obtained.

The 3D PEO/PDMS films with polygon and cylindrical helix structures were fabricated according to the above operation, including two situations: PEO layer inwards and PEO layer outwards. The atmosphere with humidity gradient was built by adding anhydrous calcium chloride in an open container and there will be a humidity gradient from the top to bottom. High humidity environment was realized by blowing wet gas.

### Fabrication of piezoelectric polymer films

To fabricate the piezoelectric film, poly(vinylidene fluoride-co-trifluoroethylene) (P(VDF-TrFE, 75/25)) powder (0.75 g, Arkema Piezotech. Co.) was dissolved into diethyl carbonate (DEC, 10 mL) under stirring at 70 °C for 2 h. After that, 3.5 mL solution was spread uniformly on a horizontal fluorosilane-modified glass sheet (10 cm × 10 cm) and dried on a heating stage at 70 °C for 30 min, 100 °C for 30 min and 135 °C for 1 h. Then, the film was separated from the glass substrate and annealed in the oven at 135 °C for 2 h to realize the transformation of β-phase^40–42^. Thus, a P(VDF-TrFE) film with the thickness of ~16 μm was obtained. Next, gold was deposited on the two sides of the plastic film at the rate of 0.4 Å s^−1^ by thermal evaporation (Nano 36, Kurt J. Lesker) with the final gold layer thickness of 70 nm, before which 5 nm chromium (Cr) was deposited at 0.1 Å s^−1^ as the adhesion layer between the gold layer and polymer film surface. Finally, the film was polarized at an electric field intensity of 50 MV/m and temperature of 100 °C for 30 min and then cooled to room temperature in the presence of the electric field^41^.

### Fabrication of 3D vibration sensors

Piezoelectric polymer films were cut into 2D precursors and then morphed into different 3D shapes by peeling-induced shape morphing. To fabricate the cantilever-like vibration sensor, a polarized P(VDF-TrFE) film was cut into belt shapes with 1.5 mm width and different lengths. The belts were morphed into spiral-like shapes by peeling from the flank of a cylinder (diameter of 4 cm) with 50 μm PDMS (silicone base: crosslinking agent = 60:1) as the adhesive layer (Supplementary Fig. 22a). The final shape was controlled by the length of the piezoelectric film and adhered parts and link belt. Several examples were shown in Supplementary Fig. 22b.

### Measurement of the vibration-sensitivity of the 3D vibration sensors

The two sides of a piezoelectric polymer film were connected to a 5 MΩ probe and the voltage change was recorded using a data acquisition system (USB-2610 Series DAQ, Smacq Technologies) in the Faraday cage. Differential mode was used during data acquisition at 10,000 Hz sampling rate and the ground terminal of the data acquisition system was connected to the Faraday cage to shield electromagnetic interference. To measure the sensitivity to vibration of different frequencies, the piezoelectric system was fixed on a vibration generator (U56001, 3B Scientific) powered by a function generator (Keysight Technologies 33210A, sinusoidal waves with amplitude of 10 V) produced periodic harmonic vibrations at programmable frequencies between 1 and 1000 Hz.

### Fabrication of 3D elastomer films

With plastic films as templates, 3D elastomer films were fabricated. Taking PDMS as an example, PDMS precursor (silicone base: crosslinking agent = 10: 1, stained in red with oil red) was spread on a PTFE film (adhered on a glass plate with certain adhesives) via blade coating. Then, the precursor was partially cured at 60 °C for 20 min and a thin PTFE film (37 μm) covered the PDMS to avoid contact with each other during subsequent process. The multilayer films were cut into pre-set structure and the 2D structures were transferred into 3D via peeling-induced shape morphing. After that, the 3D multilayer films were kept in an oven at 80 °C for 2 h and PDMS films with different 3D structures were obtained after the PTFE films were removed (Supplementary Fig. 25c). If there are transferable tattoos on the plastic film, for example Au patterns on PTFE film, the tattoos can be transferred onto the elastomer film (PDMS film, Supplementary Fig. 25d).

### SEM observation of the samples

To explore the reason for the resistance change after peeling-induced shape morphing, the Au-polymer film before and after shape morphing was observed with a scanning electron microscope (SEM).

The PLA belt was micro scale; thus, SEM was used to observe the morphologies of the shaped PLA belt.

To verify the connection between PEO and PDMS in the bilayer film, the cross-section of the film was observed with SEM.

All the above samples were observed using a field-emission SEM instrument (JEOL 7600F) after sputter coating with gold except the Au-polymer films.

### Analytic model of the cylindrical helices

Cylindrical helices can be obtained when the deviation angle is not 0° (Fig. 2f and Supplementary Fig. 16). As shown in Supplementary Fig. 16a, the cylindrical centerline is parallel to AB. OB’ is perpendicular to AB. For the film part of ABB’A’, during the shape forming after peeling, OB’ is an arc length for the cylinder and OB is the rise distance along the cylindrical centerline. Thus, the coil number (*N*) of the film part of ABB’A’ cylindrical helices can be expressed as,

8 $$N=\frac{{OB}^{\prime} }{\pi d}=\frac{{OB}}{P}$$

where *d* denotes the diameter of the cylinder and *P* the pitch of the helix. Therefore, the relationship between the pitch (*P*), diameter (*d*) and deviation angle (*δ*) can be obtained as

9 $$P=\frac{{OB}}{{OB}^{\prime} }\pi d={\rm tan\delta}\; \pi d$$

## Supplementary information

Supplementary Information

Description of Additional Supplementary Files

Supplementary Movie 1

Supplementary Movie 2

Supplementary Movie 3

Supplementary Movie 4

Supplementary Movie 5

Supplementary Movie 6

Supplementary Movie 7

Supplementary Movie 8

Supplementary Movie 9

Supplementary Movie 10

Supplementary Movie 11

Supplementary Movie 12

## Data Availability

The data supporting the findings of this study are available within the Article and its Supplementary Information. Source data are provided with this paper. Other raw data generated during this study are available from the corresponding authors upon request. Source data are provided with this paper.

## Source data

Source Data
