# Supplementary material for: Shape morphing of plastic films
Source: Nat Commun. 2022 Nov 26;13:7294. doi: 10.1038/s41467-022-34844-y (PMC9701196; doi:10.1038/s41467-022-34844-y)
Supplement: Supplementary file 1 — Supplementary Information [file 41467_2022_34844_MOESM1_ESM.pdf]

Supplementary Information

Shape morphing of plastic films

Feilong Zhang[1,6], Dong Li[2,6], Changxian Wang[1], Zhihua Liu[3], Man Yang[4], Zequn Cui[1], Junqi Yi[1], Ming Wang[1], Ying Jiang[1], Zhisheng Lv[3], Shutao Wang[4]*, Huajian Gao[2,5]*, Xiaodong Chen[1,3]*

[1]Innovative Center for Flexible Devices (iFLEX), Max Planck–NTU Joint Lab for Artificial Senses, School of Materials Science and Engineering, Nanyang Technological University, 50 Nanyang Avenue, Singapore 639798, Singapore.
[2] School of Mechanical and Aerospace Engineering, Nanyang Technological University, 50 Nanyang Avenue, Singapore 639798, Singapore.
[3]Institute of Materials Research and Engineering (IMRE), Agency for Science Technology and Research, 2 Fusionopolis Way, Innovis, #08-03, Singapore 138634, Singapore.
[4]CAS Key Laboratory of Bio-inspired Materials and Interfacial Science, CAS Center for Excellence in Nanoscience, Technical Institute of Physics and Chemistry, Chinese Academy of Sciences, Beijing 100190, P. R. China.
[5]Institute of High-Performance Computing, Agency for Science Technology and Research, 1 Fusionopolis Way, #16-16 Connexis, Singapore 138632, Singapore
[6]These authors contributed equally: Feilong Zhang, Dong Li.
*Email: stwang@mail.ipc.ac.cn (S. W.); huajian.gao@ntu.edu.sg (H. G.); chenxd@ntu.edu.sg (X. C.)

This PDF file includes:
Supplementary Text
Supplementary Figs. 1-25
Supplementary Tables. 1-2
References

Supplementary Text

Theoretical model of peeling-induced shape morphing (zero deviation angle)

The material of the plastic film is assumed to be elastoplastic with linear kinematic hardening, a common constitutive relation for polymeric and metallic materials (Supplementary Fig. 4). If we consider a material point in a tensile state, the constitutive law in terms of true stress (σ) and true strain (ε) can be expressed as

$$\sigma = \begin{cases} E\varepsilon, & 0 < \varepsilon \le \varepsilon_0, \\ H\varepsilon + (E-H)\varepsilon_0, & \varepsilon_0 < \varepsilon \le \varepsilon_B, \\ E\varepsilon + (E-H)(\varepsilon_0 - \varepsilon_B), & \varepsilon_B - 2\varepsilon_0 < \varepsilon \le \varepsilon_B, \\ H\varepsilon + (H-E)\varepsilon_0, & 0 < \varepsilon \le \varepsilon_B - 2\varepsilon_0, \\ E\varepsilon + (H-E)\varepsilon_0, & 0 < \varepsilon \le (1 - H/E)\varepsilon_0, \end{cases} \tag{S1}$$

where E and H are Young's modulus and hardening modulus of the film, respectively, while ε_0 and $\varepsilon_B > 2\varepsilon_0$ denote strains of the film at yielding (point A in Supplementary Fig. 4) and peak loading (point B in Supplementary Fig. 4). Note that when $\varepsilon_B < 2\varepsilon_0$ the reverse yielding of the film (the third part in Eq. (S1)) will not occur. The part of the constitutive law for a point in a compressive state has a similar form to equation (S1), as shown in Supplementary Fig. 4.

Since the elastic modulus of the film is much larger than that of the adhesive, deformation of the film due to interfacial interaction is considered negligible and that due to bending dominant. The bending moment M on a cross-section of the film produced by the axial stresses (Supplementary Fig. 5a) can be calculated as

$$M = \int_S y\sigma(\varepsilon)\, \mathrm{d}S, \tag{S2}$$

where $\varepsilon = ky$. Note that S is the cross-section area of the film and k the local curvature of the neutral plane of the film. For a material with the stress-strain curve in equation (S1), the corresponding moment-curvature curve (Supplementary Fig. 5b) of the film turns out to be

$$M = \begin{cases} EI_z k, & 0 < k \le \dfrac{2\varepsilon_0}{h}, \\ HI_z k + (E-H)I_z \dfrac{\varepsilon_0}{h}\left[3 - 4\left(\dfrac{\varepsilon_0}{kh}\right)^2\right], & \dfrac{2\varepsilon_0}{h} < k \le \dfrac{2\varepsilon_B}{h}, \\ EI_z k - (E-H)I_z \dfrac{\varepsilon_B}{h}\left(\dfrac{\varepsilon_0}{\varepsilon_B} + 2\right)\left(\dfrac{\varepsilon_0}{\varepsilon_B} - 1\right)^2, & \dfrac{2(\varepsilon_B - 2\varepsilon_0)}{h} < k \le \dfrac{2\varepsilon_B}{h}, \\ HI_z k + (E-H)I_z \dfrac{\varepsilon_0}{h}\left[\dfrac{32\varepsilon_0^2}{(2\varepsilon_B - kh)^2} - \dfrac{\varepsilon_0^2}{\varepsilon_B^2} - 3\right], & 0 < k \le \dfrac{2(\varepsilon_B - 2\varepsilon_0)}{h}, \\ EI_z k - (E-H)I_z \dfrac{\varepsilon_0}{h}\left(3 - \dfrac{7\varepsilon_0^2}{\varepsilon_B^2}\right), & 0 < k \le k_r, \end{cases} \tag{S3}$$

where $I_z = wh^3/12$ is the bending stiffness of the film, h and w denoting the thickness and width of the film, respectively. Besides, k_r in equation (S3) denotes the local curvature of the neutral plane of the released film. Note that the maximum positive and negative stresses occur on the two surfaces of the film ($y = \pm h/2$).

The released configuration of the film can be classified into three cases based on the value of the maximum strain ε_B over the film: (I) $\varepsilon_B \leq \varepsilon_0$ where there is no plastic deformation in the film during the peeling process; (II) $\varepsilon_0 < \varepsilon_B \leq 2\varepsilon_0$ where there is yielding but no reverse yielding during the unloading process; (III) $\varepsilon_B > 2\varepsilon_0$ where reverse yielding takes place, as shown in Supplementary Fig. 4. Among the three cases, shape morphing of the film only occurs in Case II and Case III where plastic deformation is present. Based on equation (S3), the curvature of the released film in Case III can be calculated from the condition that the moment in the released film is zero, i.e.,

$$M_r = EI_z k_r - (E - H)I_z \frac{\varepsilon_0}{h}\left(3 - \frac{7\varepsilon_0^2}{\varepsilon_B^2}\right) = 0, \tag{S4}$$

which yields

$$k_r = \frac{\varepsilon_0}{h}\left(1 - \frac{H}{E}\right)\left(3 - \frac{7\varepsilon_0^2}{\varepsilon_B^2}\right), \qquad \varepsilon_B > 2\varepsilon_0. \tag{S5a}$$

The curvature in Case II can be obtained similarly as

$$k_r = \frac{\varepsilon_B}{h}\left(1 - \frac{H}{E}\right)\left(\frac{\varepsilon_0}{\varepsilon_B} + 2\right)\left(\frac{\varepsilon_0}{\varepsilon_B} - 1\right)^2, \qquad \varepsilon_0 < \varepsilon_B \leq 2\varepsilon_0. \tag{S5b}$$

Note that in Eqs. (5a) and (5b), the maximum strain ε_B is unknown at this point. To determine ε_B, we consider energy balance in steady-state peeling of a film, as well as the equilibrium conditions of the parts of the film before and after the detachment front, respectively.

1) Energy balance in steady-state peeling

The energy balance during peeling of an elastic thin film was first developed by Kendall (1975)[1], and then extended to the cases of elastic-plastic films[2,3]. For a small element of the film $\mathrm{d}l$, we have

$$(1 - \cos\phi)P_0 w \,\mathrm{d}l = \mathrm{d}W_\mathrm{d} + \mathrm{d}W_\mathrm{r} + \Gamma^\mathrm{a} w \,\mathrm{d}l, \tag{S6}$$

where ϕ and P_0 are the peeling angle and peeling force, while W_d and W_r denote the dissipation energy and residual strain energy. The effective adhesion energy of the viscoelastic adhesive Γ^a is determined as[4-8]

$$\Gamma^\mathrm{a} = \Gamma_0^\mathrm{a}[1 + \kappa(a_T v)^n], \tag{S7}$$

where Γ_0^a is the quasi-static or intrinsic adhesion and v the propagation speed of the detachment front or approximately the peeling speed. In equation (S7), a_T is the Williams-Landel-Ferry factor associated with the temperature effect, while κ and n are materials constants of the adhesive material.

In steady-state peeling shown in Supplementary Fig. 6, the process of the detachment front propagating a small length of $\mathrm{d}l$ is equivalent to moving an element $\mathrm{d}l$ of the film in stress-free state (in the well-bonded region) along the deformed film into the long flat region TT'. Therefore, the work expenditure of this part of the film can be calculated as

$$\mathrm{d}W_\mathrm{d} + \mathrm{d}W_\mathrm{r} = \left[\int_L M(k)\,\mathrm{d}k\right] w \,\mathrm{d}l, \tag{S8}$$

where L is the path *o-a-b-c-d-e* in the M-k curve shown in Supplementary Fig. 5b. For the M-k curve in equation (S3), it can be shown that

$$\frac{\mathrm{d}W_\mathrm{d}}{\mathrm{d}l}+\frac{\mathrm{d}W_\mathrm{r}}{\mathrm{d}l}=\begin{cases}\dfrac{2(E-H)I_z\varepsilon_B^2}{h^2}\left(2\dfrac{\varepsilon_0}{\varepsilon_B}+1\right)\left(\dfrac{\varepsilon_0}{\varepsilon_B}-1\right)^2, & \varepsilon_0<\varepsilon_B\leq 2\varepsilon_0,\\ \dfrac{2(E-H)I_z\varepsilon_0\varepsilon_B}{h^2}\left(10\dfrac{\varepsilon_0^2}{\varepsilon_B^2}-15\dfrac{\varepsilon_0}{\varepsilon_B}+6\right), & \varepsilon_B>2\varepsilon_0.\end{cases}$$

(S9a, b)

Combining Eqs. (S6), (S7) and (S9), we have when $\varepsilon_0<\varepsilon_B\leq 2\varepsilon_0$,

$$(1-\cos\phi)P_0w=\Gamma_0^\mathrm{a}[1+\kappa(a_Tv)^n]w+\frac{2(E-H)I_z\varepsilon_B^2}{h^2}\left(2\frac{\varepsilon_0}{\varepsilon_B}+1\right)\left(\frac{\varepsilon_0}{\varepsilon_B}-1\right)^2,$$

(S10a)

and when $\varepsilon_B>2\varepsilon_0$,

$$(1-\cos\phi)P_0w=\Gamma_0^\mathrm{a}[1+\kappa(a_Tv)^n]w+\frac{2(E-H)I_z\varepsilon_0\varepsilon_B}{h^2}\left(10\frac{\varepsilon_0^2}{\varepsilon_B^2}-15\frac{\varepsilon_0}{\varepsilon_B}+6\right).$$

(S10b)

2) Analysis of the peeled-off part of the film after the detachment front

In Supplementary Fig. 6, the dashed line QR represents a plane that is tangent to the film on point Q, then the peeled-off part of the film can be regarded as peeled from a rigid plane with a very thin adhesive whose thickness is negligible. This problem for an elastic film has been studied by Kendall (1973)[9]. For simplicity, we adopt Kendall's theory and did not consider the plasticity of the film which has a weak effect in this part. Numerical results in Supplementary Fig. 7 confirm the feasibility of this simplification.

From Supplementary Fig. 6, we can obtain the relation

$$\theta_l=\frac{\pi}{2}-\phi_1=\frac{\pi}{2}-\phi+\phi_0. \tag{S11}$$

Kendall's theory shows that $l^2=2EI_z(1-\sin\theta_l)/(P_0w)$, which, combined with equation (S11), yields

$$l^2=\frac{2EI_z}{P_0w}[1-\cos(\phi-\phi_0)]. \tag{S12}$$

With equation (S12), the bending moment at the cross-section Q can be approximated as

$$M_Q=P_0wl=\sqrt{2EI_zP_0w[1-\cos(\phi-\phi_0)]}. \tag{S13}$$

Since the constitutive law of the film has been assumed to be elastic, the curvature at Q can be obtained as

$$k_Q=\frac{M_Q}{EI_z}. \tag{S14}$$

The location of the largest curvature in the film can be approximately considered to be at Q, so the strain ε_B is reached on the inner surface (the one bonded to the adhesive) of the film at Q. Therefore, using equation (S14) we have

$$\varepsilon_B = \frac{k_Q h}{2} = \frac{M_Q h}{2EI_z}. \tag{S15}$$

3) Analysis for the adherent part of the film before detachment front

The part of the film before detachment front in Supplementary Fig. 6 can be regarded as a beam on an elastic foundation (Winkler foundation), without considering the plasticity of the film for the sake of simplicity. The governing equation for such problem of a linear elastic beam is[10-12]

$$EI_z \frac{\mathrm{d}^4 y}{\mathrm{d}x^4} + K^a y = 0, \tag{S16}$$

with K^a being the stiffness of the foundation. For an adhesive with elastic modulus E^{a} and thickness t, we have

$$K^a = \frac{E^{\mathrm{a}} w}{t}. \tag{S17}$$

By applying boundary conditions

$$y \to 0, \qquad \frac{\mathrm{d}y}{\mathrm{d}x} \to 0 \qquad \text{as } x \to \infty, \tag{S18a, b}$$

$$EI_z \frac{\mathrm{d}^2 y}{\mathrm{d}x^2} = M_Q, \qquad EI_z \frac{\mathrm{d}^3 y}{\mathrm{d}x^3} = 0 \qquad \text{at } x = 0, \tag{S18c, d}$$

we can obtain the solution to equation (S16) as

$$y = \frac{M_Q}{2EI_z c^2} e^{-cx} (\cos cx - \sin cx), \tag{S19}$$

with the constant

$$c = \left(\frac{K^a}{4EI_z}\right)^{1/4}. \tag{S20}$$

Therefore, the slope of the film at Q is

$$\tan\phi_0 = \frac{M_Q}{EI_z c} = \frac{M_Q}{EI_z}\left(\frac{4EI_z t}{E^a w}\right)^{1/4}. \tag{S21}$$

Now we can combine equations (S10, S13, S15, and S21) to solve for the value of ε_B, and then use equation (5a and 5b) to calculate the curvature of the released film. The peeling force can also be obtained using equation (S10).

Analyzing the applicable range of thickness and modulus in bilayer films consisting of an elastic layer and a plastic layer for peeling-induced shape morphing strategy

Further, we theoretically analyzed the applicable range of thickness and modulus in each layer. Assume that the elastic layer has the thickness h_1 and elastic modulus E_1, and the plastic layer has the thickness h_2, elastic modulus E_2, and yield strain ε_{02}. Consider the critical case in which the maximum strain (absolute value) in the plastic layer during peeling right reaches ε_{02}, i.e.,

$$\varepsilon_{\max} = k\frac{h_1 + h_2}{2} = \varepsilon_{02} \tag{S22}$$

where k is the curvature of the film. The bending moment on cross-section of the film

can be calculated as

$$M=\int_{-\frac{h_1+h_2}{2}}^{\frac{h_1-h_2}{2}} E_1 k h^2 w \mathrm{d}h+\int_{\frac{h_1-h_2}{2}}^{\frac{h_1+h_2}{2}} E_2 k h^2 w \mathrm{d}h$$

$$=\frac{E_1 h_1 k w}{12}\left(h_1^2+3 h_2^2\right)+\frac{E_2 h_2 k w}{12}\left(3 h_1^2+h_2^2\right) \quad (\mathrm{S}23)$$

where w is the width of the film. In Eq. (S23), h_1 is assumed to be no less than h_2, and similar results can be obtained for the other situations. To simplify this discussion, we consider the case in which the deformation of the adhesive layer can be neglected and the angle ϕ_0 in Supplementary Fig. 6 is zero. Therefore, Eq. (S13) can be written as

$$M=\sqrt{\frac{P_0 \bar{E} w^2 h_{\mathrm{f}}^3}{6}(1-\cos \phi)} \qquad (S24)$$

where P is the peeling force, ϕ the peeling angle, $h_{\mathrm{f}}=h_1+h_2$ the film thicknessand $\bar{E}=\left(E_1 h_1+E_2 h_2\right) / h_{\mathrm{f}}$ the averaged elastic modulus of the film. From Eqs. (S22)-(S24), we obtain

$$\varepsilon_{\max }=\frac{h_{\mathrm{f}}^2 \sqrt{6 P_0 E h_{\mathrm{f}}(1-\cos \phi)}}{E_1 h_1\left(h_1^2+3 h_2^2\right)+E_2 h_2\left(3 h_1^2+h_2^2\right)}. \qquad (S25)$$

Here we consider a special case with $E_1=E_2$ and $h_1=h_2$, and Eq. (S25) becomes

$$\varepsilon_{\max }=\sqrt{\frac{3 P_0(1-\cos \phi)}{E_1 h_1}}. \qquad (S26)$$

To induce plastic deformation in the plastic layer, we need $\varepsilon_{\max } \geq \varepsilon_{02}$ which, combined with Eq. (S22), leads to

$$E_1 h_1 \leq \frac{3 P_0(1-\cos \phi)}{\left(\varepsilon_{02}\right)^2}. \qquad (S27)$$

Eq. (S27) gives us a rough evaluation for the range of layer thickness and elastic modulus in some specific cases ($E_1=E_2$ and $h_1=h_2$). However, a full analysis for this problem is a very challenging task and will be included in our future research plans.

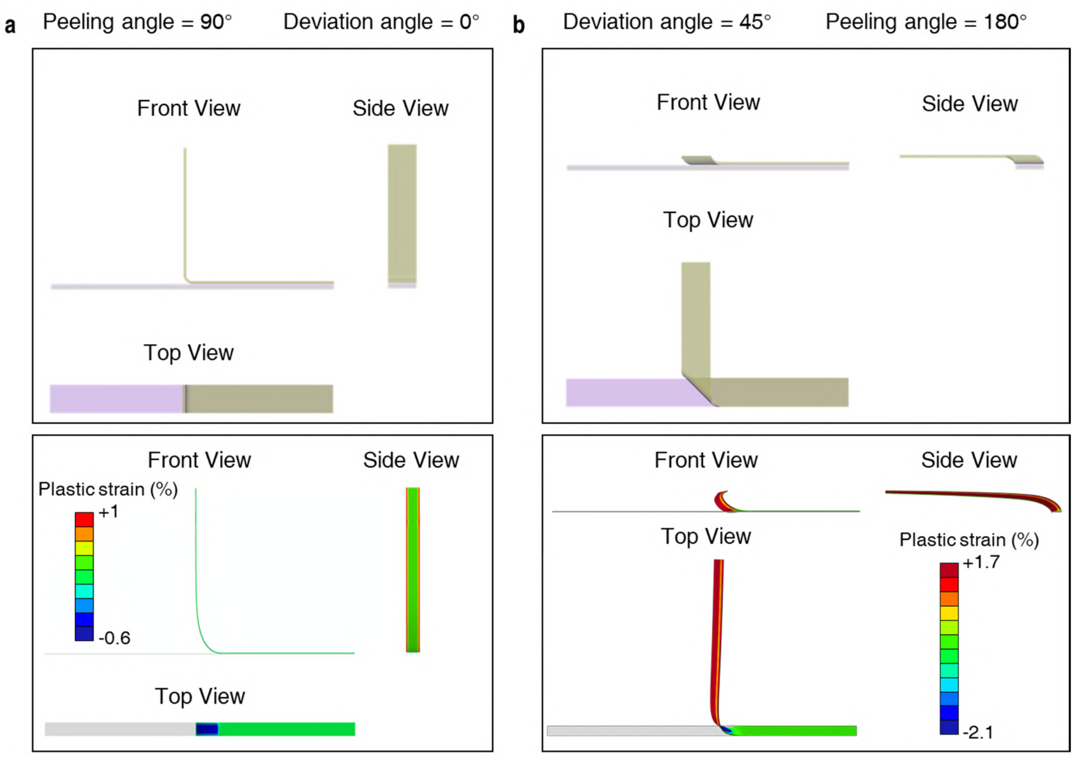

Supplementary Fig. 1. Three-view drawing of a film under peeling. a, A film subject to a peeling angle of 90°. **b**, A film peeled in the direction perpendicular to the long axis of the film with a peeling angle of 180° and a deviation angle of 45°. Top: 3D models of the peeling process; bottom: the results of FEA.

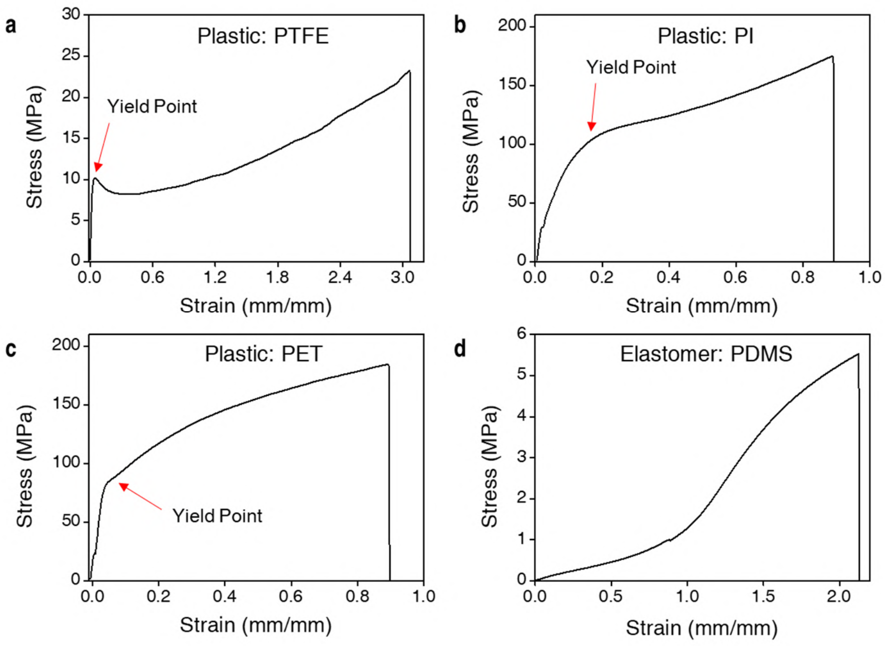

Supplementary Fig. 2. Stress-strain curves of plastic and elastomer films. a-c, Plastic films with yield points: (a) PTFE; (b) PI; (c) PET. **d**, Elastomer film, PDMS.

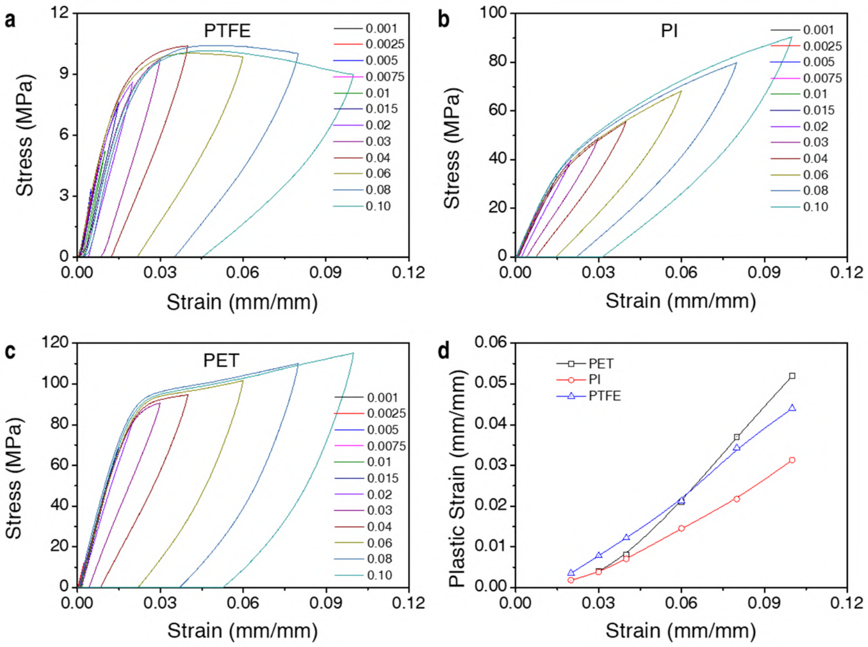

Supplementary Fig. 3. Plastic strain of plastic films. a-c, Stress-strain hysteresis loops of plastic films at different strains: (a) PTFE; (b) PI; and (c) PET. **d**, Plastic strain of plastic films at different strains. Source data are provided as a Source Data file.

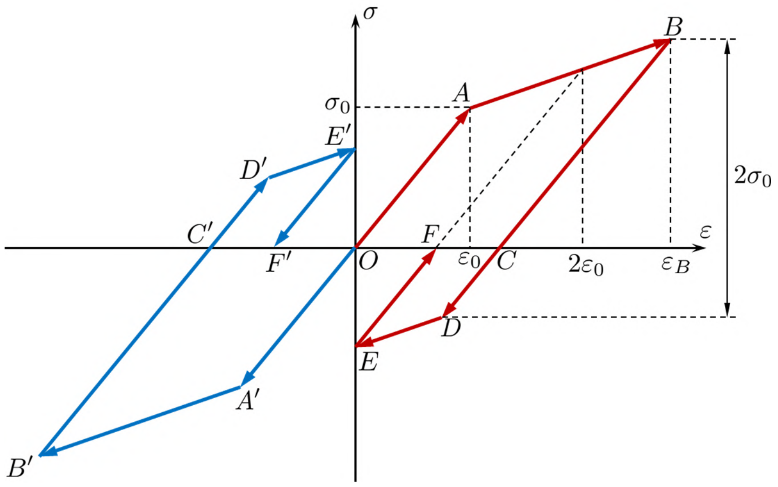

Supplementary Fig. 4. Constitutive law of the film material adopted in the mechanical peeling model. The red arrows represent a cycle of tensile loading (*OA, AB*) → unloading (*BC*) → reverse loading (*CD, DE*) → unloading (*EF*) on the film, while blue arrows represent a cycle starting from compressive loading. It can be proven that when $\varepsilon_B > 2\varepsilon_0$, there is a reverse yielding stage *DE*, corresponding to Case III; when $\varepsilon_0 < \varepsilon_B \leq 2\varepsilon_0$, the reverse loading is elastic, and the stage *DE* is degenerated, corresponding to Case II.

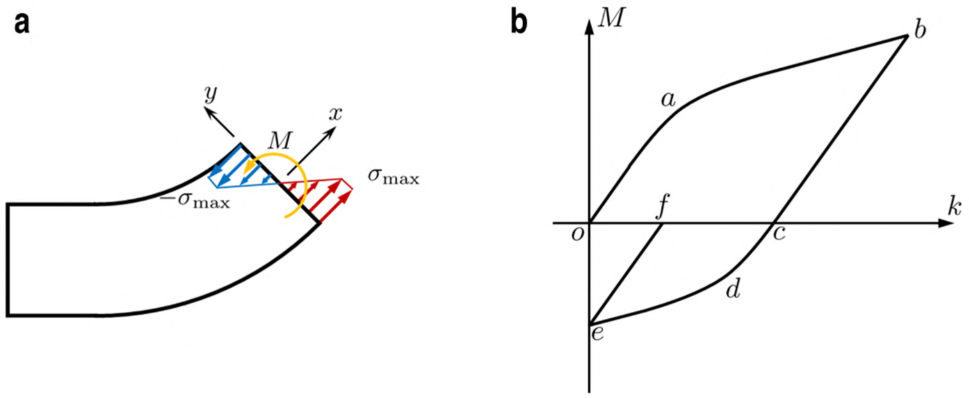

Supplementary Fig. 5. Bending deformation of the film. a, Bending moment *M* produced by axial stresses (red and blue arrows) on the cross-section of the film. **b**, Moment-curvature relation for a film with the constitutive law shown in Supplementary Fig. 3. The moment-curvature curve shows a cycle of bending (*oa, ab*) → unloading (*bc*) → reverse bending (*cd, de*) → unloading (*ef*) of the film.

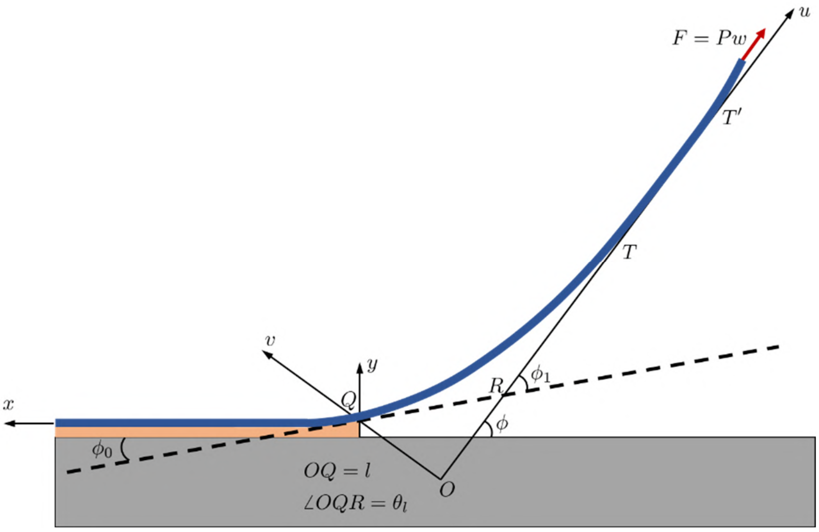

Supplementary Fig. 6. The steady-state peeling configuration of a plastic film attached to a rigid substrate. For the detached part of the film, the system of coordinate uOv is created with the v axis crossing the detachment front Q and the u axis coincided with the long flat part of the film TT' along the loading direction. For the attached part of the film, the horizontal x axis is along the undeformed thin film, while vertical y axis passes through the detachment front Q. The dashed line QR is tangent to the thin film at Q.

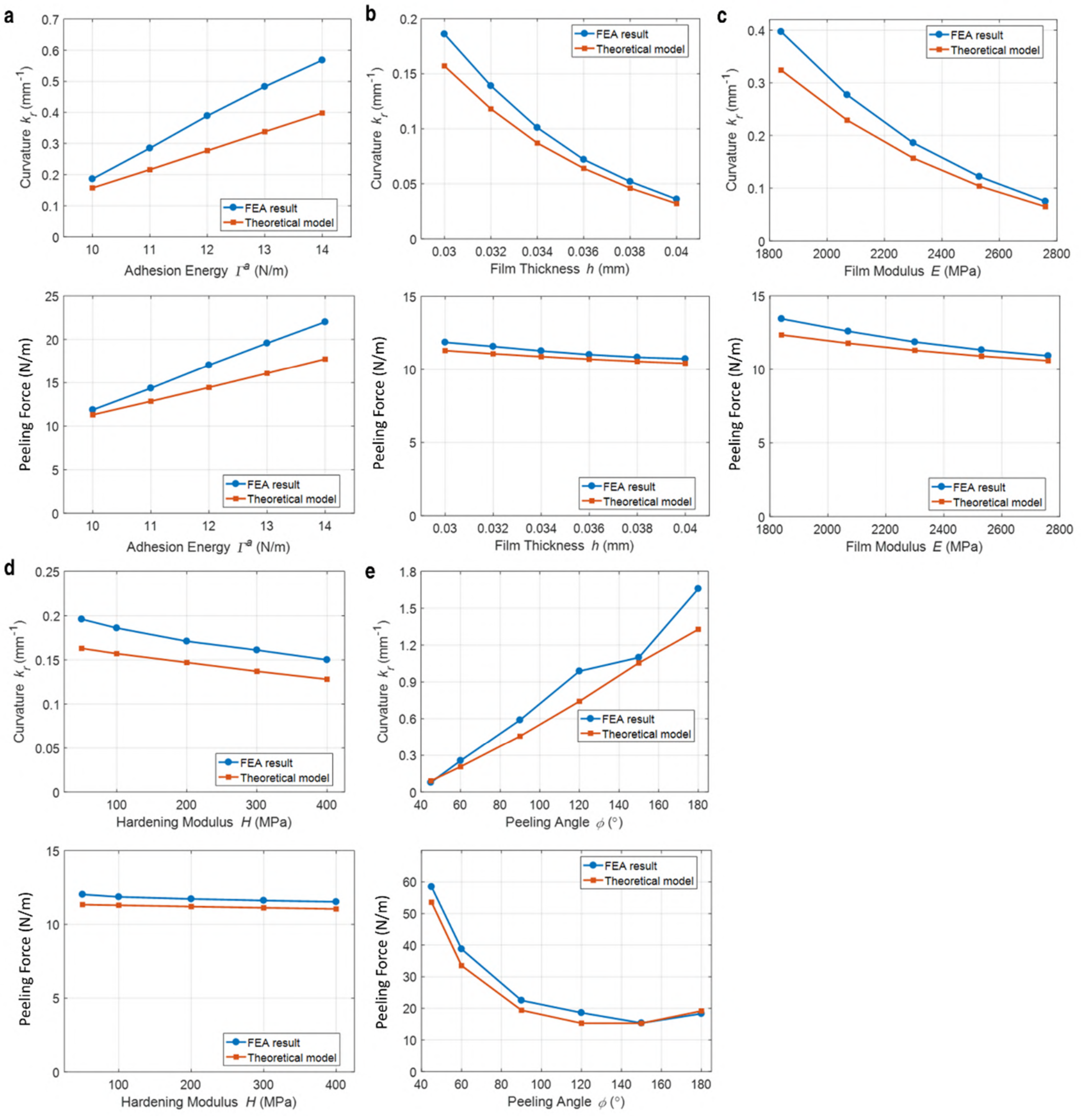

Supplementary Fig. 7. Comparison of results from finite element analysis and theoretical model on the curvature of the released film (first row) and the force in steady-state peeling (second row) with varying parameters. a, effective adhesion energy Γ^a of the adhesive; **b**, thickness h of the film; **c**, Young's modulus E of the film (the yield strain of the film is kept the same in all cases); **d**, hardening modulus H of the film; **e**, peeling angle ϕ. Source data are provided as a Source Data file.

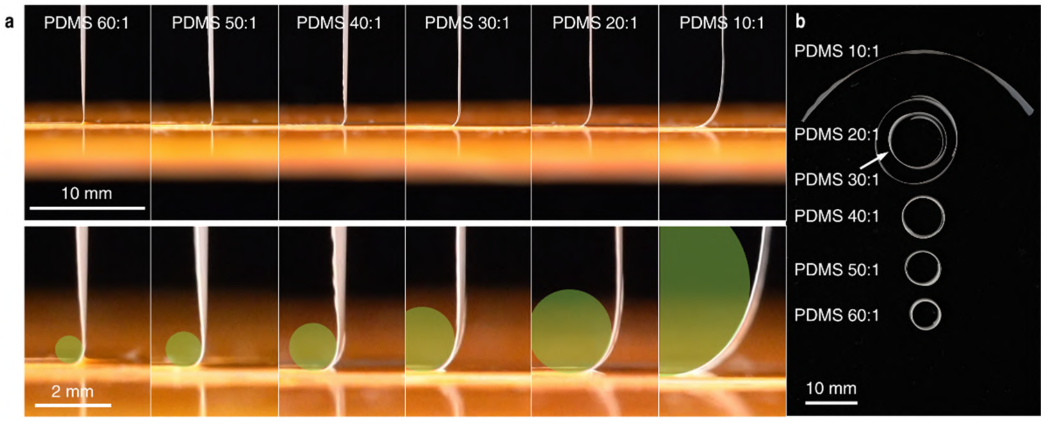

Supplementary Fig. 8. Peeling of PTFE film with adhesives of different interface fracture energy. a, Optical images of the detached region during peeling experiments with PTFE thickness of 37 μm, peeling angle of 90º, peeling speed of 10 mm/s and adhesive layer thickness of 56 μm. The green shadows indicate the stickier adhesive leads to larger bending degree of the plastic film. **b**, Pictures of the bent films after being peeled off from the adhesives with different interface fracture energy.

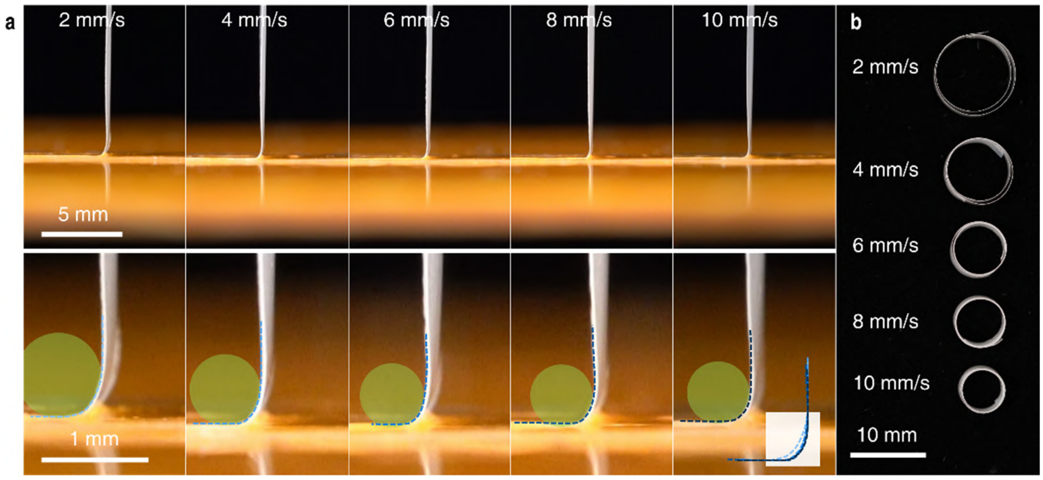

Supplementary Fig. 9. Peeling of PTFE film with different peeling speeds. a, Optical images of the detached region during peeling experiments with PTFE thickness of 37 μm, peeling angle of 90º, PDMS (60:1) as adhesive and adhesive layer thickness of 56 μm. The dash lines (deepening blue with the increase of peeling speeds) illustrate the outline of the bending part of the film during peeling and the inset is the assembly of the outlines indicating the curvature of the bending films during peeling increased with the peeling speeds. **b**, Pictures of the bent films after being peeled off with different peeling speeds.

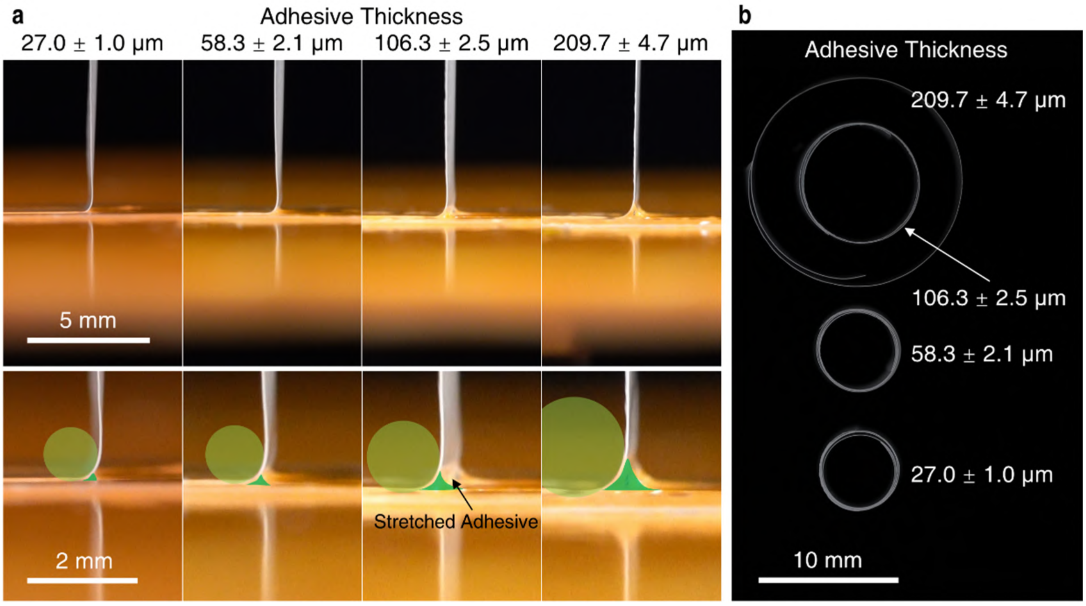

Supplementary Fig. 10. Peeling of PTFE film with different adhesive thicknesses. **a**, Optical images of the detached region during peeling experiments with PTFE thickness of 37 μm, peeling angle of 90°, PDMS (60:1) as adhesive and peeling speed of 10 mm/s. As the increase of the adhesive layer, a larger volume of adhesive is subjected to deformation in the detached region. The dark green shadows indicate the involved adhesive. **b**, Optical images of the bent films after being peeled off with different adhesive thicknesses showing that thicker adhesive leads to smaller curvature.

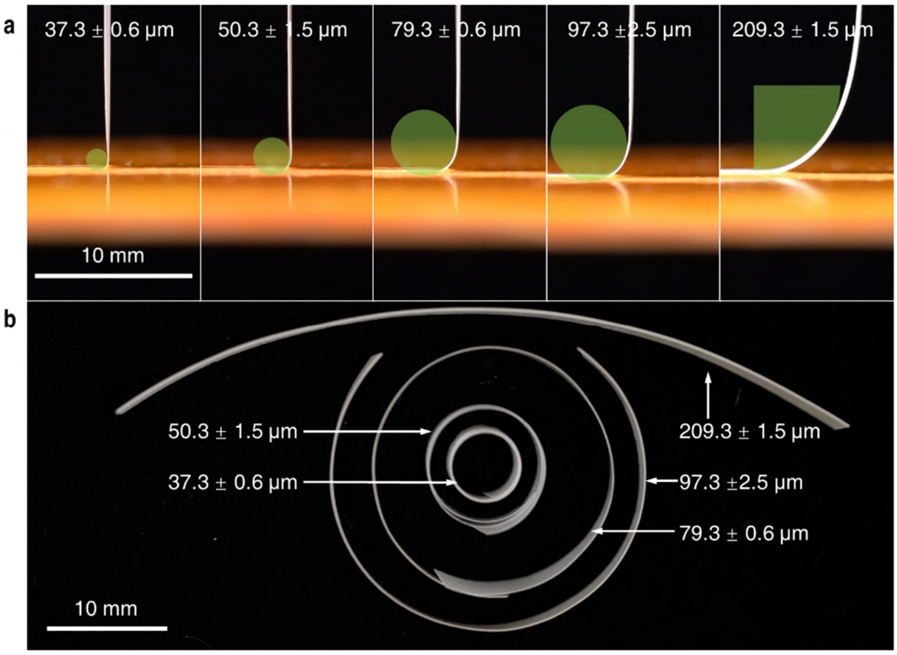

Supplementary Fig. 11. Peeling of PTFE film with different thicknesses. a, Optical images of the detached region during peeling experiments with peeling speed of 10 mm/s, peeling angle of 90º, PDMS (60:1) as adhesive and adhesive layer thickness of 56 µm. The green shadows indicate that thicker plastic film leads to smaller bending degree during peeling. **b**, Optical images of the bent films after being peeled off with different thicknesses showing that thicker plastic film leads to smaller curvature.

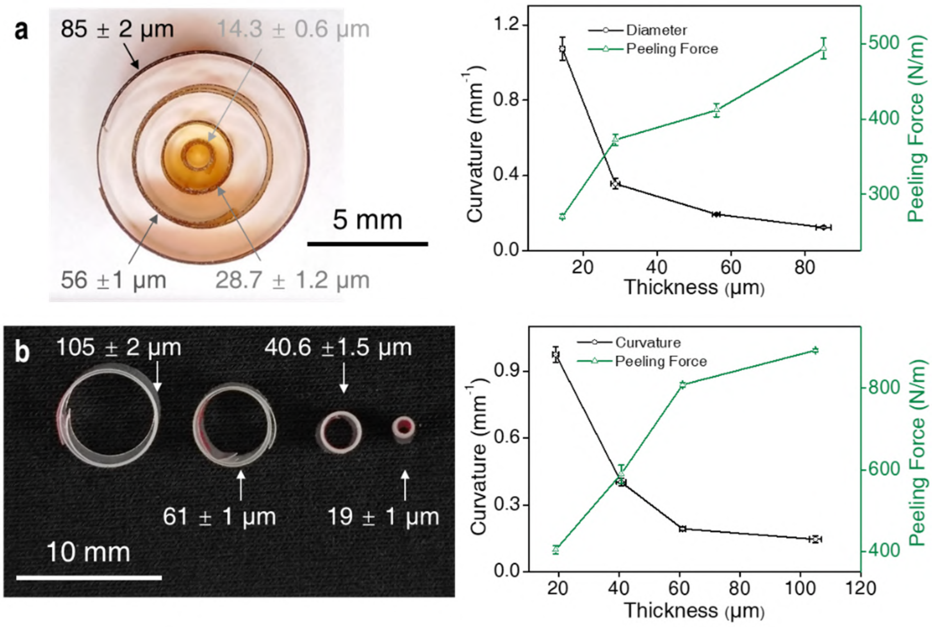

Supplementary Fig. 12. The peeled-off PI and PET films with different thicknesses at peeling speed of 10 mm/s, peeling angle of 180° and commercial Kapton Tape as the adhesive layer. a, The curvature of the peeled-off PI films decreased with the film thickness. **b**, The curvature of the peeled-off PET films decreased with the film thickness. In a and b, left is the optical images of the peeled films and the arrows indicate the film thickness; right is the graphs of curvature of the peeled film and peeling force verse the film thickness, indicating thicker film leads to smaller curvature. The data are presented as mean ± s.d.. Source data are provided as a Source Data file.

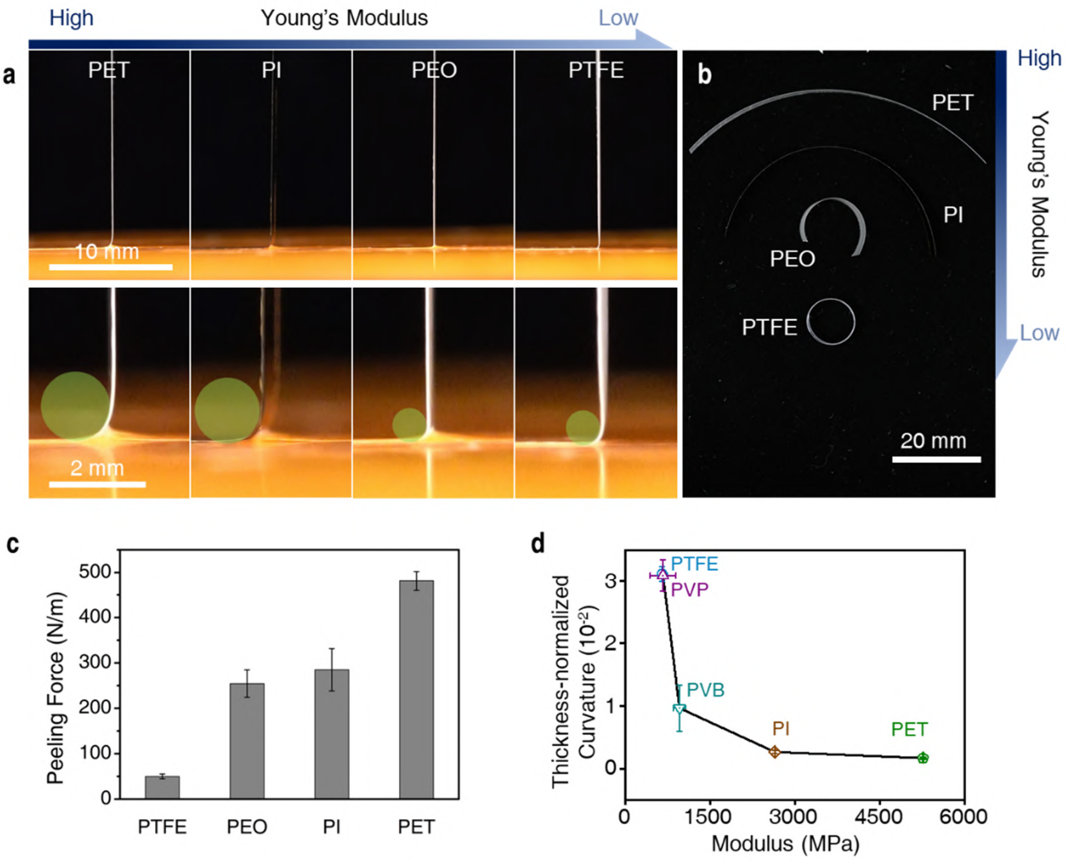

Supplementary Fig. 13. Peeling of films with different modulus. **a**, Optical images of the deadhesion region during peeling experiments of different plastic films with the film thickness of 50-60 μm, peeling speed of 10 mm/s, peeling angle of 90°, and commercial Kapton Tape as the adhesive layer. Green shadows indicate the film with high modulus was hard to bend during peeling. **b**, Optical images of the bent films after being peeled off showing films with high modulus lead to smaller curvature. **c**, Peeling force during peeling of the films with different modulus. **d**, The thickness-normalized curvature of plastic films with different modulus (PTFE, PVP, PVB, PI, and PET) shows that stiffer films with high modulus are unfavorable for shape morphing. The data in c and d are presented as mean ± s.d.. Source data are provided as a Source Data file.

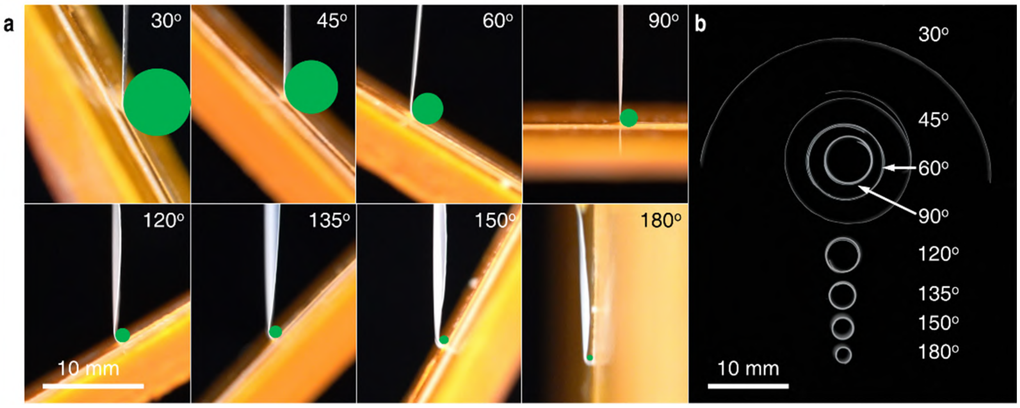

Supplementary Fig. 14. Peeling of PTFE film at different peeling angles. a, Optical images of the detached region during peeling experiments with PTFE thickness of 37 μm, peeling speed of 10 mm/s, PDMS (60:1) as adhesive and adhesive layer thickness of 56 μm. Green circles indicate larger peeling angles lead to larger bending degrees of the plastic film during peeling. **b**, Optical images of the bent films after being peeled off showing that peeling at large peeling angles results in large curvature.

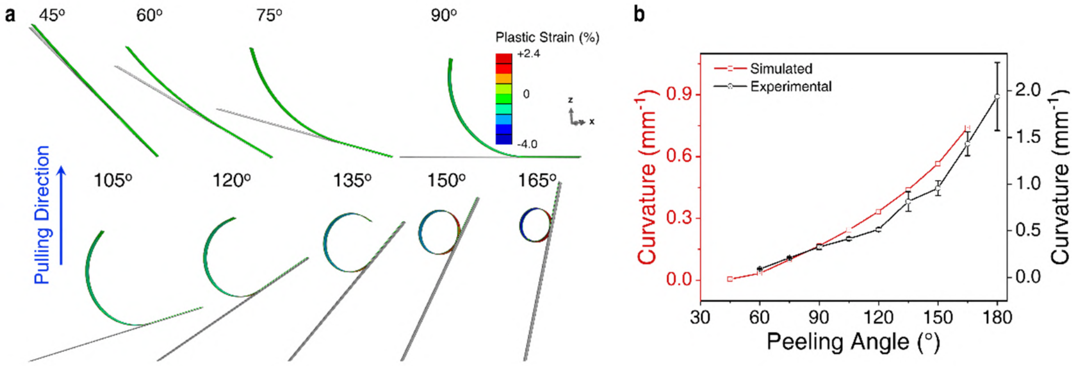

Supplementary Fig. 15. The effect of peeling angle on the curvature of peeled plastic PI film. a, FEA results of PI films (30 µm thick, 5 mm wide) peeled off from Kapton tape at different peeling angles. **b**, Graph shows larger peeling angles leading to larger curvature of the peeled PI films. The experimental results and the FEA results exhibit similar tendency and are in reasonable agreement. The data are presented as mean ± s.d.. Source data are provided as a Source Data file.

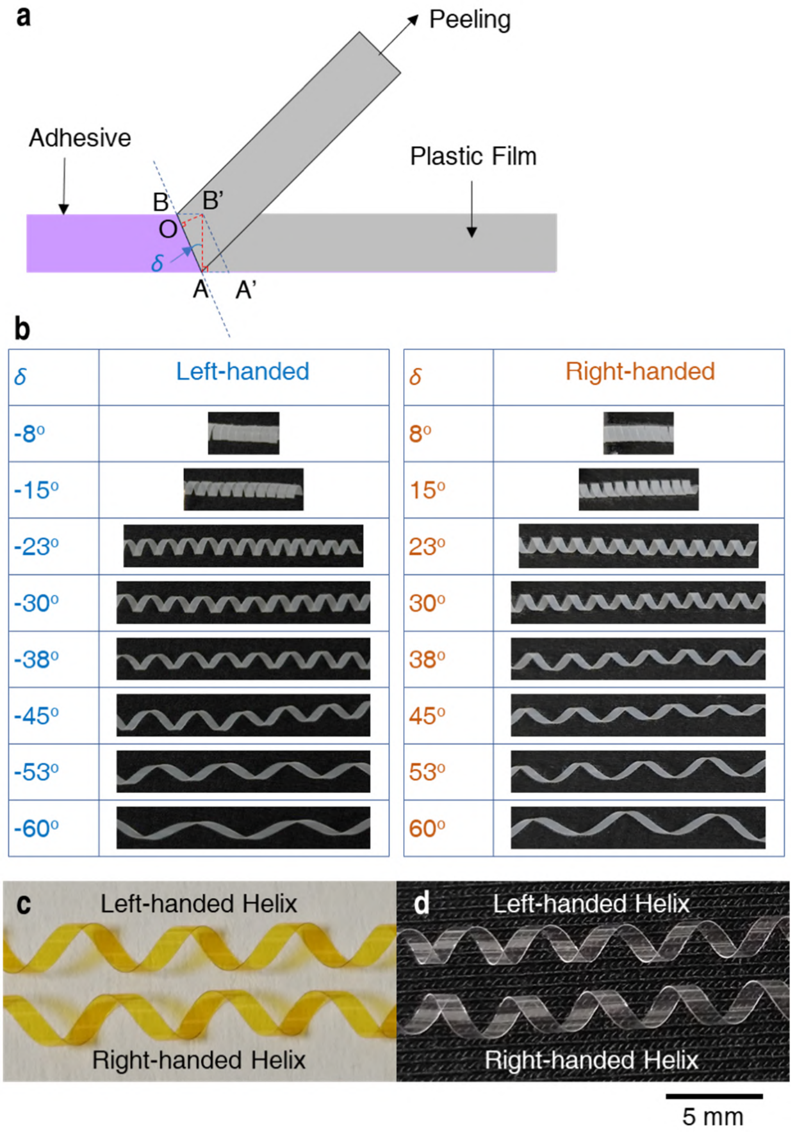

Supplementary Fig. 16. The helical structures by peeling at different deviation angles with peeling speed of 10 mm/s, peeling angle of 180° and commercial Kapton Tape as the adhesive layer. a, Top-view schematic illustrating the parameters during peeling at certain deviation angle (δ). **b**, Photographs of the PTFE (80 μm) helical films peeled at deviation angles from -60° to 60°. **c**, The helices of PI films (thickness of 28.7 ± 1.2 μm) with different chirality peeled at δ of 45° and -45°. **d**, The helices of peeled PET films (thickness of 40.6 ±1.5 μm) with different chirality peeled at δ of 45° and -45°.

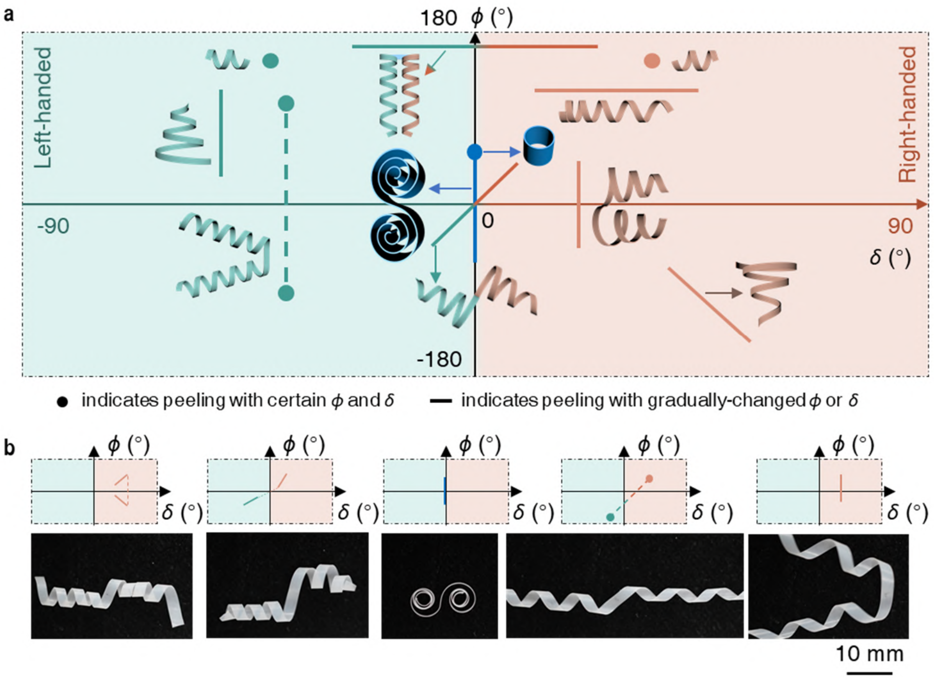

Supplementary Fig. 17. 3D structures from 2D belts obtained by programming the peeling process. a, Phase–like diagram of the peeling-induced 3D structures and the peeling process with ϕ and δ. The negative peeling angle indicates the other surface of the film was adhered. The dots indicate peeling at a certain peeling angle and deviation angle. The lines indicate peeling at gradually changing peeling angles and deviation angles. The dashed line connecting the dots or lines means a multi-step peeling process. The final 3D structures can be predicted according to the previous conclusion by changing the peeling angle and deviation angle. **b**, Several 3D structures from 2D belts and the corresponding peeling process.

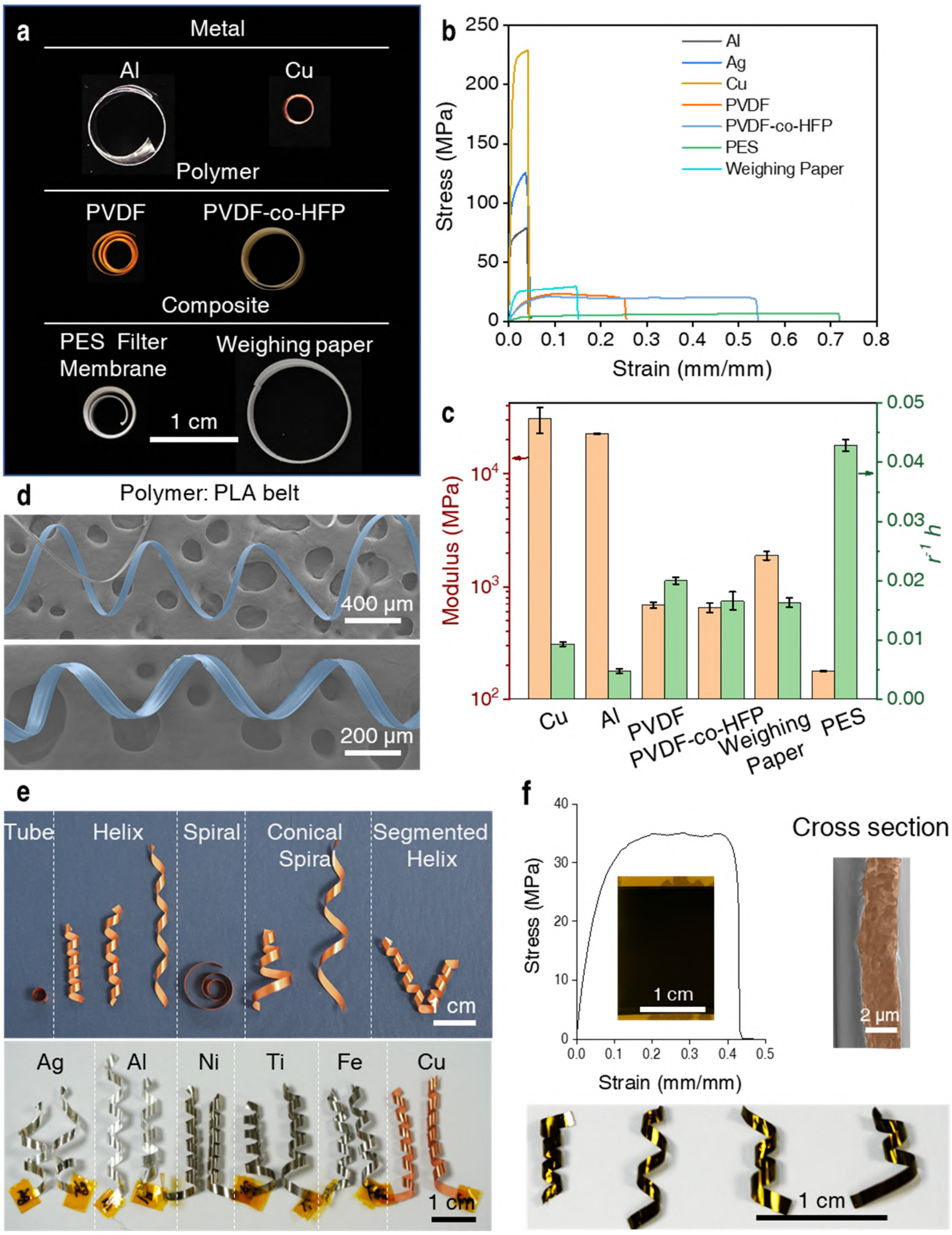

Supplementary Fig. 18. Peeling-induced shape morphing is applicable to various plastic materials. a, Peeling-induced tubes of metal, polymer, and composite films. **b**, Representative stress-strain curves of the films verifying their plasticity. **c**, The thickness-normalized curvature and modulus of these plastic films. The data are presented as mean ± s.d.. **d**, Peeling-induced shape morphing of poly lactic acid (PLA) at the microscale. **e**, The 3D structures of metal films by peeling-induced shape morphing. Top: 3D structures of copper film (adhesive: PDMS (50:1)); bottom: helical structures of metals films including silver (Ag), aluminum (Al), nickel (Ni), titanium (Ti), and iron (Fe). **f**, The 3D structures of semiconductor films ((poly(3-hexylthiophene-2,5-diyl), P3HT with thickness of ~2 µm, modulus of 399.6 ± 39.6 MPa, and yield strain of ~0.1) by peeling-induced shape morphing. Error bars represent standard deviation. Source data are provided as a Source Data file.

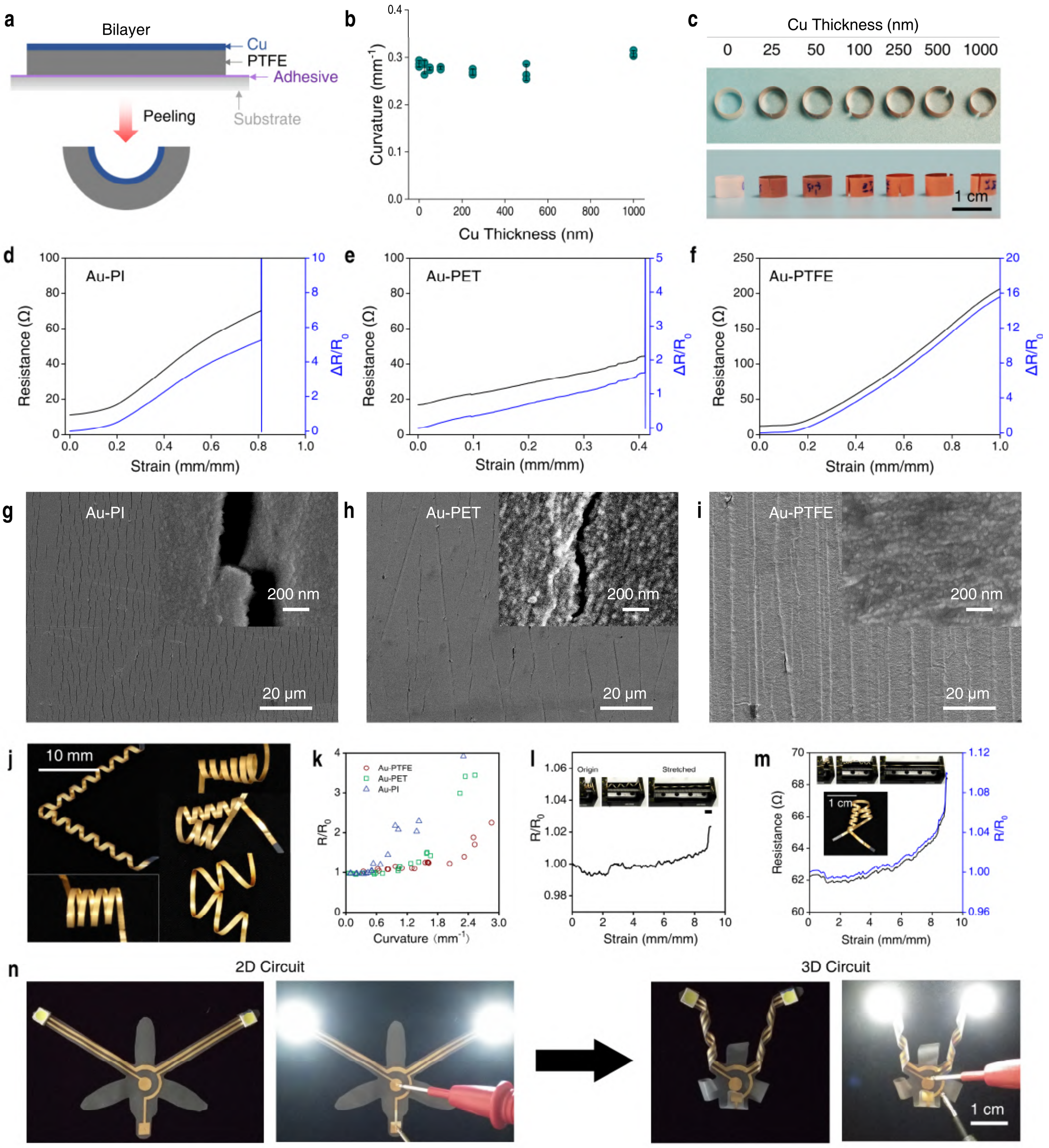

Supplementary Fig. 19. Peeling-induced shape morphing of metal-polymer bilayer film. a-c, The effect of metal thickness on the shape morphing. Polymer film: PTFE (~90 μm); metal: copper (Cu). a, Schematics showing the PTFE-Cu bilayer film and the peeling process. b, The curvature of the peeled bilayer film versus the Cu layer thickness. c, Optical images of the peeled bilayer film. **d-f**, Resistance-strain curves of the Au (70 nm)-plastic films: (d) Au-PI; (e) Au-PET; (f) Au-PTFE. **g-i**, The SEM image of the Au on the plastic films after peeling-induced shape morphing: (g) Au-PI with curvature of ~1.2 mm^{-1}; (h) Au-PET with curvature of ~0.7 mm^{-1}; (i) Au-PTFE with curvature of ~1.4 mm^{-1}. Insets showing the cracks of the Au layer caused by the shape morphing which induces the increase of the resistance. **j**, Different 3D complex structures of the Au-PTFE films via peeling-induced shape morphing. **k**, Graph shows the resistances of the Au-polymer films decrease first and then increase with the increase of curvature, indicating the successive compression and stretching of the Au layer. Even under rather large curvature (< 2.3 mm-1 for Au-PTFE, < 1.5 mm-1 for Au-

PET, and < 0.8 mm-1 for Au-PI), the gold layer in the peeled Au-PI, Au-PET and Au-PTFE films showed only a slight increase (< 40%) in resistance caused by discrete micro-cracks formed from stretching the gold layer along the plastic film. **l**, Relative resistance-strain curve of the peeling-induced Au-PTFE cylindrical helix shows resistance remained nearly unchanged when helices are stretched. Inset: photographs showing stepwise stretching of Au-PTFE cylindrical helices using a dynamic tensile stage. **m**, Resistance-strain curves of the Au-PTFE films (width of 1 mm) with tendril-like structure by peeling-induced shape morphing showing the tendril-like Au-PTFE film can be stretched to 9 times its original length without significant change in resistance. **n**, Photographs of 3D circuits (left) obtained from 2D circuits (right) by peeling-induced shape morphing functioned equally well, showing shape-morphing is potentially a simple strategy for fabricating 3D electronics. Error bars represent standard deviation. Source data are provided as a Source Data file.

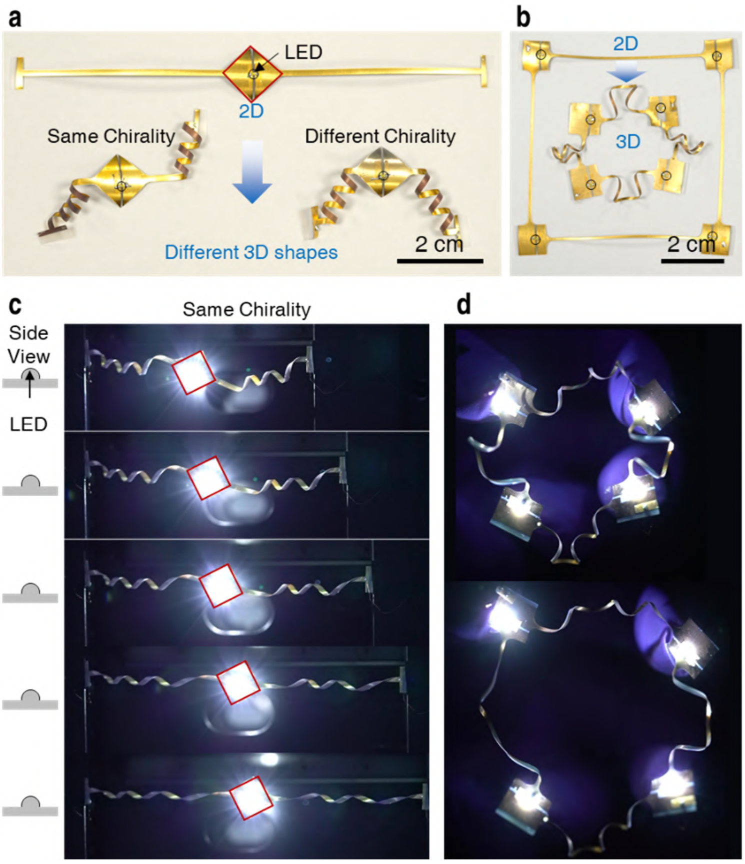

Supplementary Fig. 20. 3D circuits from the 2D ones by peeling-induced shape morphing strategy. a, A 3D circuits with a LED connected by two helical wires from the 2D one. Left: two wires with left-handed helix; right: two helical wires with different chirality. **b**, A closed-loop circuits with 4 LEDs connected by the helical wires from 2D one. **c**, The stretching processes of the circuits in (a). The circuit showed good stretchability without compromising the light intensity. **d**, The circuit with 4 LED on 4 fingertips can change their shapes freely.

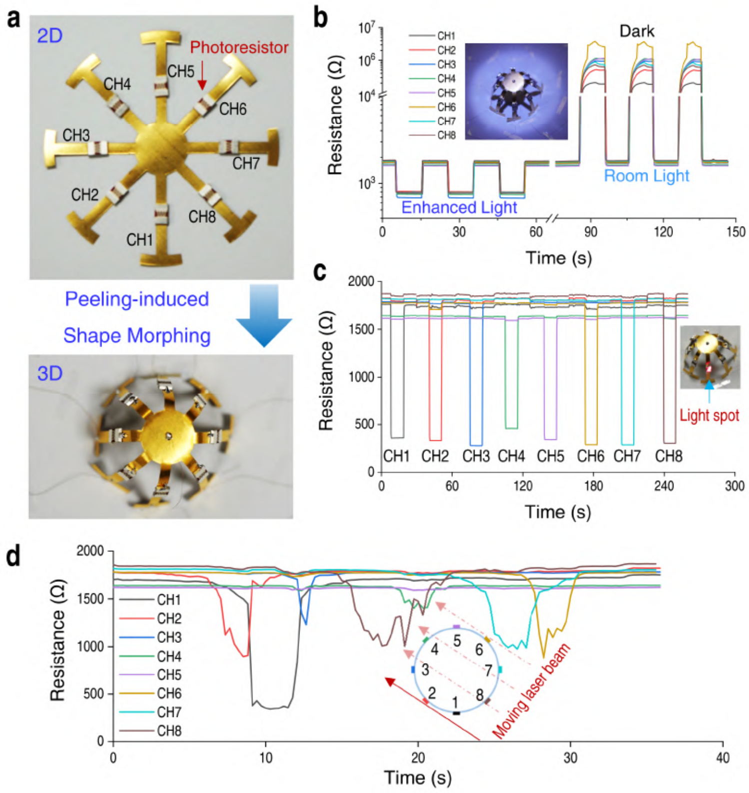

Supplementary Fig. 21. A 3D circuits accommodating eight photoresistors for photodetection. a, Photos of the 2D circuits with photoresistors at eight directions and the 3D circuits by peeling-induced shape morphing. CH1-CH8 indicate the eight channels with photoresistors. **b**, The resistance changes of the 8 photoresistors with the intensity variation of the environment: dark, room light and enhanced light. **c**, The single photoresistor perceives the local light with decreased resistance due to the increase of light intensity. **d**, The perception of the 3D photodetector to the moving laser beam. Source data are provided as a Source Data file.

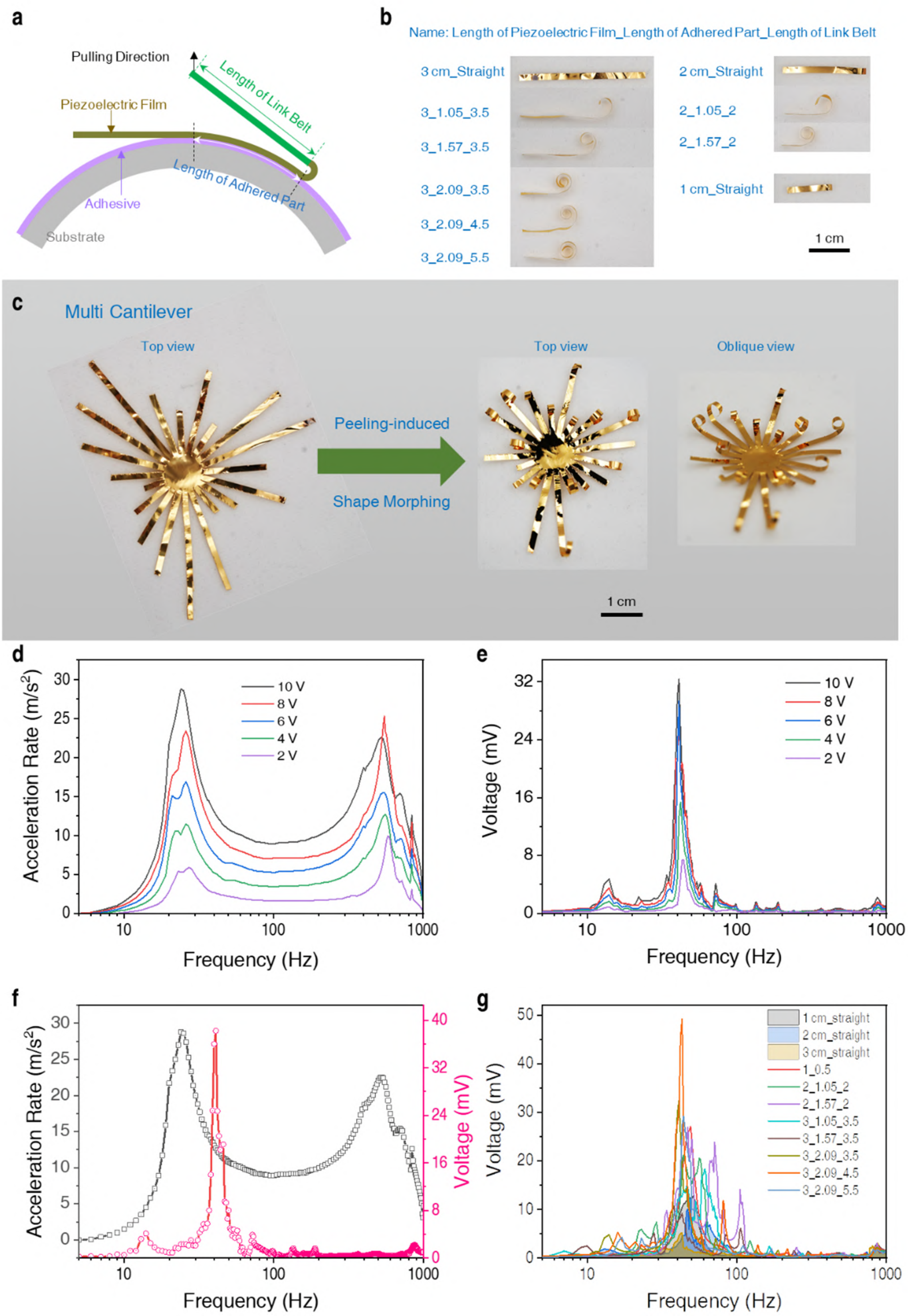

Supplementary Fig. 22. Peeling-induced shape morphing in piezoelectric systems. **a**, Schematic showing front view of the peeling process. The morphologies of the peeled piezoelectric films were controlled by the length of the film, the adhered parts and the link belt. **b**, The photographs of the films after peeling-induced shape morphing. **c**, The photographs of the multi-cantilever piezoelectric system. **d**, High voltages induced larger acceleration rate of the vibration generator. **e**, Larger acceleration rate enhanced the generated voltage of the cantilever (3_2.09_3.5). **f**, The acceleration rate of the vibration generator and the generated voltage of the cantilever under different

frequency, indicating a resonance at 41 Hz of the cantilever (3_2.09_3.5). **g**, The electrical output characteristics of the piezoelectric system with different structures. After peeling-induced shape morphing, the 3D cantilever can sense more broad vibrational frequency (24-92 Hz with voltage > 1 mV for 3_1.05_3.5) and generate higher voltage (peak voltage 53.4 mV for 3_2.09_4.5) than the 2D precursor film (1.5 mm × 3 cm, 27-53 Hz with voltage > 1 mV, peak voltage 5.7 mV). Source data are provided as a Source Data file.

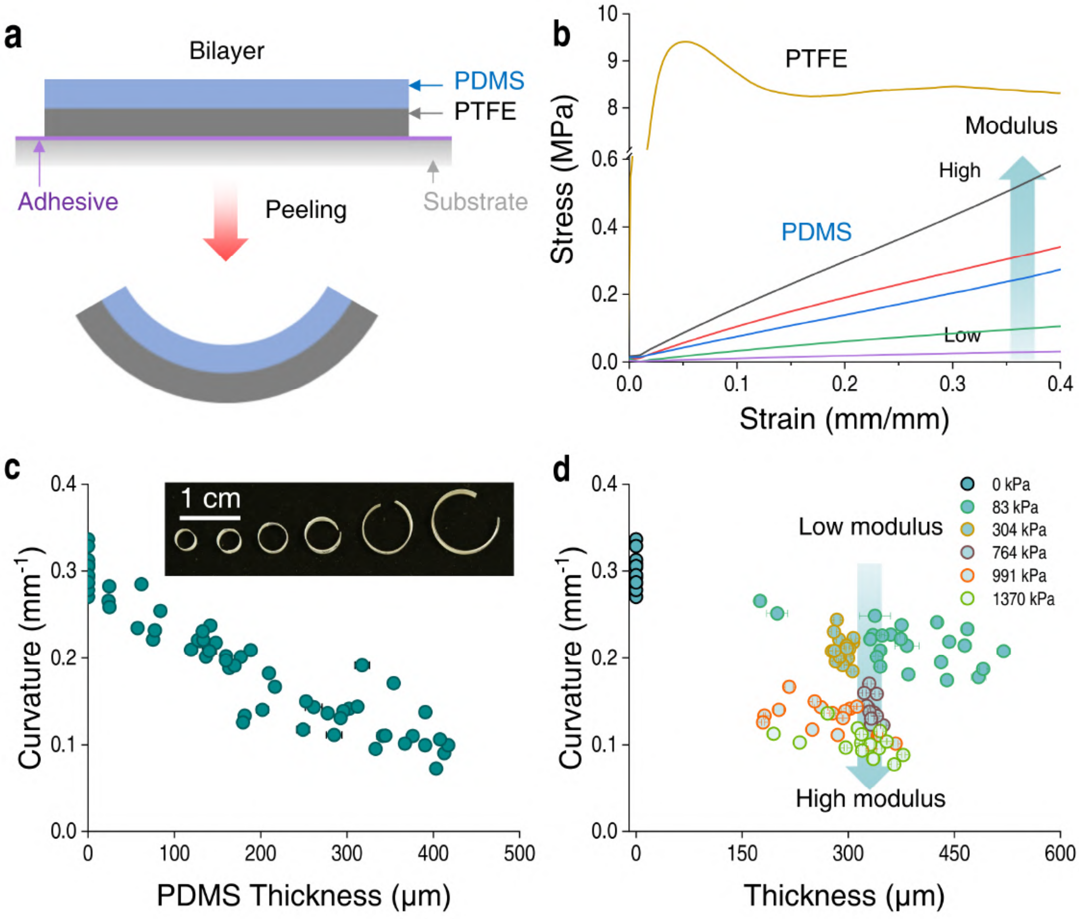

Supplementary Fig. 23. Peeling-induced shape morphing of plastic film-elastomer bilayer film. a, Schematics showing the PTFE-PDMS bilayer film and the peeling process. **b**, The stress-strain curves of PTFE and PDMS with different modulus. **c**, The curvature of the peeled PTFE-PDMS bilayer films versus the PDMS thickness. Inset: Optical images of the peeled PTFE-PDMS bilayer films with different PDMS thickness. **d**, The effect of PDMS modulus on the curvature of the peeled PTFE-PDMS bilayer films. Source data are provided as a Source Data file.

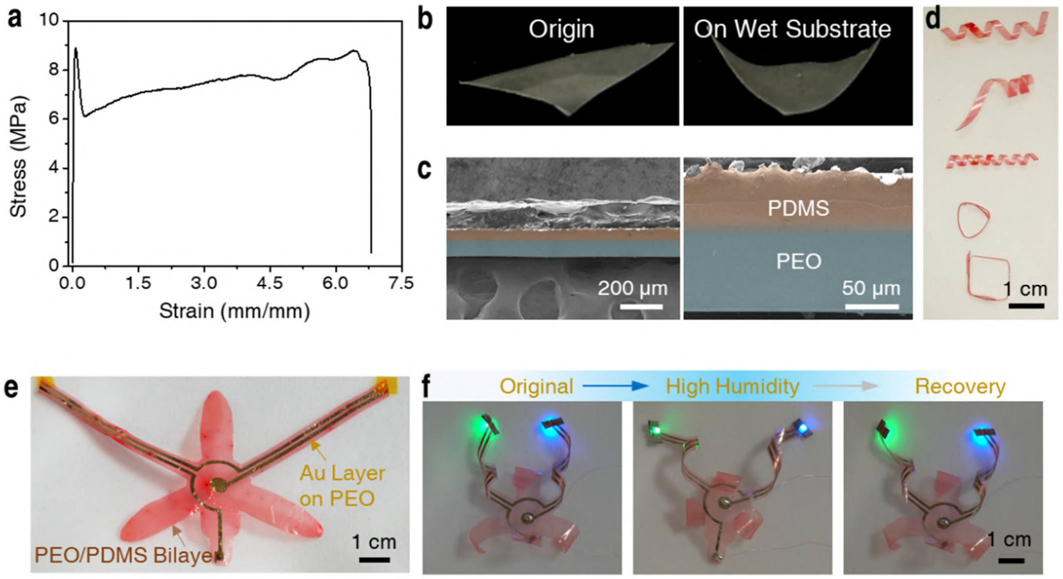

Supplementary Fig. 24. Peeling-induced shape morphing of bilayer film of active PEO and PDMS. a, Stress-strain curves of plastic PEO films showing the plasticity of PEO films with small yield strain and large elongation at break. **b**, The humidity responsive PEO film. The PEO film bent away from the wet substrate. **c**, The cross-section view of the bilayer film of active PEO and PDMS after pseudocolor processing. **d**, Different 3D complex structures of the bilayer film *via* peeling-induced shape morphing due to the plasticity of the PEO layer. **e**, Circuits on the PEO/PDMS bilayer film. **f**, Active 3D circuits. The 3D circuit on PEO/PDMS film kept working when the shape changed under changing humidity, indicating the potential application in 4D electronics. Source data are provided as a Source Data file.

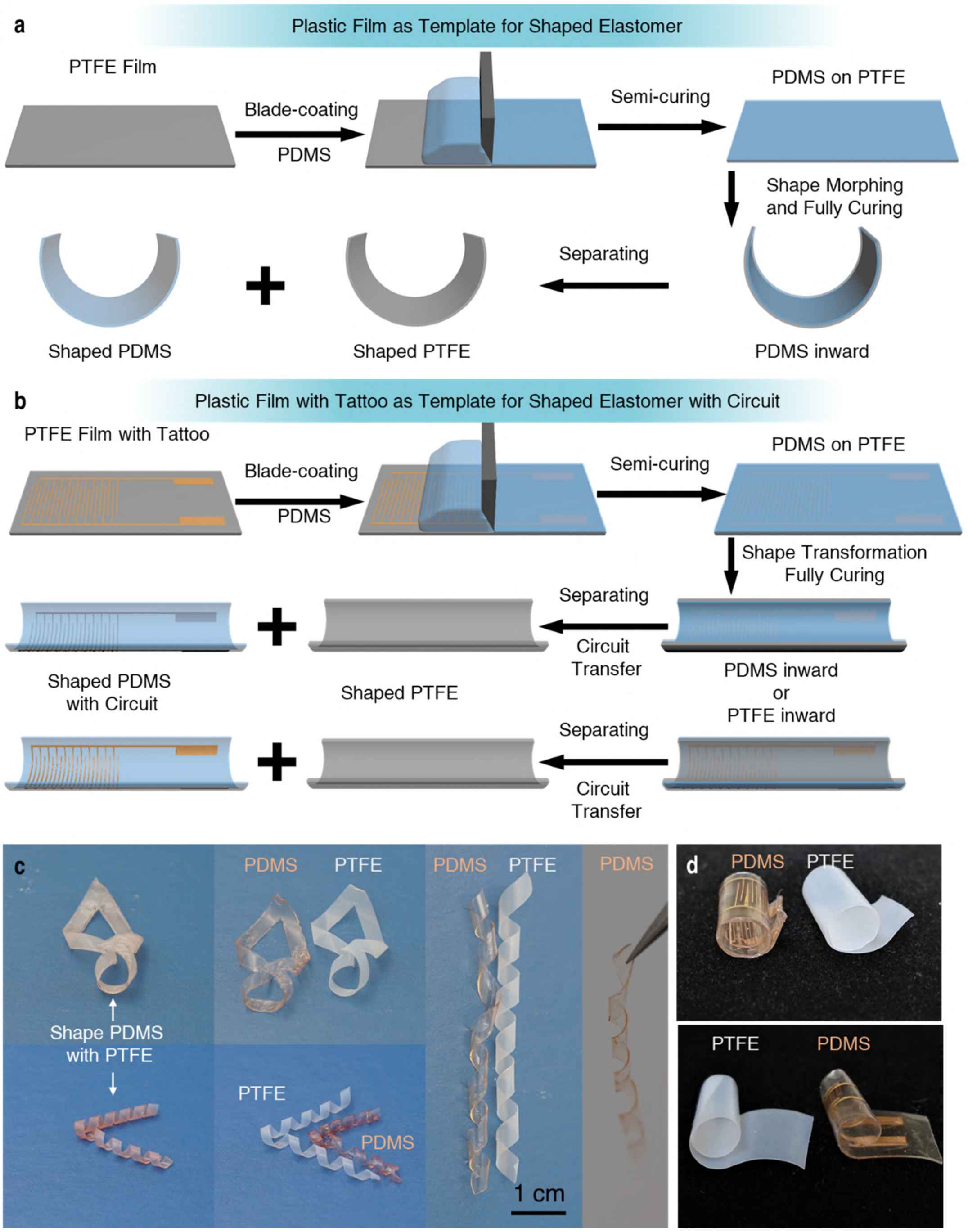

Supplementary Fig. 25. Fabrication of 3D elastomer films with plastic film as a template. a-b, Schematics showing the fabrication of Pure 3D PDMS (a) and 3D PDMS with Au circuits transferred from plastic film (b) by peeling-induced shape morphing. PDMS precursor was spread on a PTFE film (adhered on a glass plate with certain adhesives) via blade coating. Then, the precursor was partially cured at 60 °C for 20 min (a thin PTFE film was covered the PDMS to avoid contact with each other during the subsequent process). The multilayer films were cut into pre-set structure and the 2D structures were transferred into 3D *via* peeling-induced shape morphing. After that, the 3D multilayer films were kept in an oven at 80 °C for 2 h and PDMS films with different 3D structures were obtained after the PTFE films were removed. If there are transferable tattoos on the plastic film, for example Au patterns on PTFE film, the

tattoos can be transferred onto the elastomer film (PDMS film). By changing the bending direction, the position of the tattoos on the curved PDMS (on the concave side or on the convex side) can be tuned. **c**, Photos of several 3D PDMS films with PTFE as substrate using peeling-induced shape morphing strategy. **d**, Photos of several 3D PDMS films with transferred Au tattoo. indicating the possibility to form fully functional 3D stretchable electronics.

Supplementary Table 1 | Parameters used in theory and finite element analysis

Symbol	Typical value	Description
E	2300 MPa	Young's modulus of the film
μ	0.35	Poisson's ratio of the film
ε_0	0.01957	Yield strain of the film
H	100 MPa	Hardening modulus of the film
h	0.03 mm	Film thickness
E^a	0.025 MPa	Young's modulus of the adhesive layer
K^a	25 MPa/mm	Normal traction-separation stiffness
Γ^a	0.01 MPa·mm or 10 N/m	Effective adhesion energy of the adhesive layer
t	0.001 mm†	Thickness of the adhesive layer (in theory)

† Note that the thickness of cohesive element layer in FEA is zero or a small number (e.g., 1.0×10^{-5} mm), while the thickness of the adhesive layer is explicitly considered in our theoretical model. In FEA, the effect of the adhesive layer thickness is incorporated in K^a via Eq. (S17).

Supplementary Table 2 | Strategies inducing plastic strain in uniform films

**Methods**	**Materials**	**Mechanism**	**Strain**	**Shape change**	**Size**	**Possible Damage**	**Film thickness**	**Ref.**
Mechanical loading apparatus	Materials with plasticity	Stretching	Uniform	Elongation or compression				[13]
Focused Ion Beam (FIB)	Single and polycrystalline solids	Interstitials and vacancies introduced during the ion beam bombardment	Asymmetric along the thickness	Bending or folding	Nano to micrometer	Damaging the crystalline state and surface structures	Tens of nanometer	[14,15]
Folding origami	Materials with plasticity	Local bending during folding	Locally asymmetric along the thickness,	Folding	macroscale	Local large strain		[16,17]
Peeling-induced shape morphing	Materials with plasticity	Bending and stretching during peeling	Asymmetric along the thickness	Bending	Micrometer to macroscale	Negligible	Micrometer to millimeter	This work

Note: Using mechanical loading apparatus can easily introduce plastic strains in films, which however are uniform and only cause elongation or compaction but not shape transformation.

Focused ion beam (FIB) can cause asymmetric plastic strain in films even at nanometer scale irradiated by the high-energy ions. However, the FIB method is limited to single and polycrystalline films with thickness of tens of nanometers and is not applicable to thick films and large-scale patterns. What's more, the FIB method commonly damages the crystalline state and surface structures of the thin films.

Folding origami mainly induces local asymmetrical plastic strain by mechanical folding the film and the residual plastic strain keeps the sharp folds along the predefined crease lines. Therefore, it is difficult for folding origami method to form smooth curved surfaces.

Our peeling-induced shape morphing strategy is applicable to most of the film materials with plasticity (homogeneous films and composite films) with the thickness from hundreds of nanometers to millimeter and lateral sizes ranging from meters to tens of micrometers. This strategy brings negligible damage to film and the curvature of peeled films can be regulated precisely.

Reference

1 Kendall, K. Thin-film peeling-the elastic term. *J. Phys. D* **8**, 1449-1452 (1975).

2 Kim, K. S. & Aravas, N. Elastoplastic analysis of the peel test. *Int. J. Solids Struct.* **24**, 417-435 (1988).

3 Aravas, N., Kim, K. S. & Loukis, M. J. On the mechanics of adhesion testing of flexible films. *Mater. Sci. Eng. A* **107**, 159-168 (1989).

4 Maugis, D. & Barquins, M. in *Adhesion and Adsorption of Polymers* (ed Lieng-Huang Lee) 203-277 (Springer US, 1980).

5 Muller, V. M. On the theory of pull-off of a viscoelastic sphere from a flat surface. *J. Adhes. Sci. Technol.* **13**, 999-1016 (1999).

6 Persson, B. N. J. & Brener, E. A. Crack propagation in viscoelastic solids. *Phys. Rev. E* **71**, 036123 (2005).

7 Greenwood, J. A. & Johnson, K. L. Oscillatory loading of a viscoelastic adhesive contact. *J. Colloid Interface Sci.* **296**, 284-291 (2006).

8 Wahl, K. J., Asif, S. A. S., Greenwood, J. A. & Johnson, K. L. Oscillating adhesive contacts between micron-scale tips and compliant polymers. *J. Colloid Interface Sci.* **296**, 178-188 (2006).

9 Kendall, K. The shapes of peeling solid films. *J. Adhes.* **5**, 105-117 (1973).

10 Yurenka, S. Peel testing of adhesive bonded metal. *J. Appl. Polym. Sci.* **6**, 136-144 (1962).

11 Gardon, J. L. Peel adhesion. II. A theoretical analysis. *J. Appl. Polym. Sci.* **7**, 643-665 (1963).

12 Kinloch, A. J., Lau, C. C. & Williams, J. G. The peeling of flexible laminates. *Int. J. Fract.* **66**, 45-70 (1994).

13 Kanik, M. *et al.* Strain-programmable fiber-based artificial muscle. *Science* **365**, 145-150 (2019).

14 Chalapat, K. et al. Self-organized origami structures via ion-induced plastic strain. *Adv. Mater.* **25**, 91-95 (2013).

15 Liu, Z. et al. Nano-kirigami with giant optical chirality. *Sci. Adv.* **4**, eaat4436 (2018).

16 Callens, S. J. P. & Zadpoor, A. A. From flat sheets to curved geometries: Origami and kirigami approaches. *Mater. Today* **21**, 241-264 (2018).

17 Dudte, L. H., Vouga, E., Tachi, T. & Mahadevan, L. Programming curvature using origami tessellations. *Nat. Mater.* **15**, 583 (2016).
