# Supplementary material for: Shape morphing of plastic films
Source: Nat Commun. 2022 Nov 26;13:7294. doi: 10.1038/s41467-022-34844-y (PMC9701196; doi:10.1038/s41467-022-34844-y)
Supplement: Supplementary file 2 — Description of Additional Supplementary Files [file 41467_2022_34844_MOESM2_ESM.docx]

**Description of Additional Supplementary Files**

**File Name: Supplementary Movie 1
Description:** Finite-element analyses of peeling-induced shape morphing. Full 3D finite-element analyses (FEAs) were adopted to analyze the peeling-induced shape morphing of 2D films. In case 1, a plastic film was peeled with a peeling angle of 90°; in case 2, the plastic film was peeled perpendicular to the long axis with peeling angle of 180°, as shown by the multi-view of the film during peeling. The plastic film is assigned properties of PI (thickness of 30 μm, width of 5 mm and length of 5 cm) with the elastic modulus (E) and Poisson’s ratio (ν) of 2.3 GPa and 0.35, respectively. The adhesive layer takes the properties of a commercial Kapton tape. The normal adhesion force and shear force between the plastic film and adhesive tape are 0.52 MPa and 0.53 MPa, respectively.

**File Name: Supplementary Movie 2
Description:** Process of peelinginduced shape morphing. A PI film with thickness of 30 μm adhered on a flat plate with Kapton tape as the adhesive layer. In case 1, the plastic film was peeled at a peeling angle of 90° and peeling speed of 10 mm/s, resulting in the formation of a circle; in case 2, the plastic film was peeled perpendicular to the long axis with a peeling angle of 180° and peeling speed of 10 mm/s, leading to a helix.

**File Name: Supplementary Movie 3
Description:** Fabrication of spirallike structure. Spiral structure formed by peeling the PTFE film (80 μm thick, 2 mm wide) from an arcuate surface with Kapton tape as the adhesive layer during which the peeling angle gradually changed as in Fig. 3a.

**File Name: Supplementary Movie 4
Description:** Fabrication of cylindrical helices with gradually increasing pitch Cylindrical helices with gradually increasing pitch formed by peeling the PTFE film (80 μm thick, 2 mm wide) from Kapton tape under gradually increasing deviation angles (peeling angle of 180° and peeling speed of 10 mm/s) as in Fig. 3b.

**File Name: Supplementary Movie 5
Description:** Fabrication of cylindrical helices with different chirality Cylindrical helices with different chirality formed by peeling the PTFE film (80 μm thick, 2 mm wide) from Kapton tape with deviation angles changing from negative to positive values (peeling angle of 180° and peeling speed of 10 mm/s) as in Fig. 3c.

**File Name: Supplementary Movie 6
Description:** Fabrication of conical spirals Conical spirals formed by peeling the PTFE film (80 μm thick, 2 mm wide) from Kapton tape when simultaneously changing peeling angles and deviation angles. In this process, the substrate with adhesive is a stationary arcuate surface and the plastic film is adhered at an angle to the centerline axis of the arcuate substrate as in Fig. 3d.

**File Name: Supplementary Movie 7
Description:** Fabrication of polygon (Triangle) Right triangle with an interior angle of 60° and shortest side of 10 mm by peeling the PTFE film (80 μm thick, 5 mm wide) from arranged adhesive layers (Kapton tape) with predetermined widths and intervals according to the lengths and interior angles of the targeted shape. With the peeling angle of 180° and peeling speed of 10 mm/s, the widths of adhesive tapes are 2.62 mm, 1.57 mm, 2.09 mm, and 2.62 mm with intervals of 17.3 mm, 10 mm and 20 mm, respectively as in Fig. 3e.

**File Name: Supplementary Movie 8
Description:** Fabrication of hyperboloid-like structure Hyperboloids were fabricated by a two-step peeling process with orthogonal peeling directions. In step I, the PTFE film (80 μm thick, 4 cm × 2 cm) was attached on two separated adhesive tapes (1 cm × 2 cm with an interval of 2 cm) and peeled off with peeling angle of 180°; in step Ⅱ, the other side of the PTFE film was adhered on the adhesive tape (2 cm × 2 cm) and peeled off at the peeling angle of 180°, before which the parts adhered in the first step were cut into parallel strips along their curling direction as in Fig. 3f.

**File Name: Supplementary Movie 9
Description:** Peeling-induced shape morphing of circuits A 2D circuit on PTFE film was fabricated via thermal evaporation of Au and LEDs were inserted in the circuit. Then, the circuit was connected to a source of direct current and the LEDs were activated. On the basis of peelinginduce shape morphing strategy, the 2D circuit was transformed into 3D orchid-like circuit and the 3D circuit worked as well as the 2D one as in Supplementary Fig. 19.

**File Name: Supplementary Movie 10
Description:** Humidity-sensitive peeling-induced polygons A PEO/PDMS bilayer film with a width of 3 mm was programmed to form a triangular structure with PEO layer outward by peeling-induced shape morphing. A transparent tank with anhydrous calcium chloride at the bottom created a humidity gradient (lower humidity at the bottom and higher humidity at the top). Due to the humidity sensitivity of the PEO layer, the peeledoff triangle of PEO/PDMS transformed into an ellipse-like shape at high humidity and into quadrilateral, pentagon and hexagon in turn when it moved gradually to a dry environment as in Supplementary Fig. 21f.

**File Name: Supplementary Movie 11
Description:** Humidity-sensitive peeling-induced cylindrical helices Two PEO/PDMS bilayer films with a width of 3 mm were programmed to form cylindrical helices structure with PEO layer outward and PEO layer inward by peeling-induced shape morphing, respectively. A transparent tank with anhydrous calcium chloride at the bottom created a low humidity environment. When transferred into a dry environment, the PEO/PDMS cylindrical helices with PEO layer inward become tight with an increase in curvature, while the one with PEO layer outward becomes loose with a decrease in curvature. Similarly, when blown by wet gas, the PEO/PDMS cylindrical helices with PEO layer inward become loose, while the one with PEO layer outward become tight as in Supplementary Fig. 21g.

**File Name: Supplementary Movie 12
Description:** Active peelinginduced 3D circuits A 3D circuit on PEO/PDMS bilayer film was fabricated based on the peeling-induced shape morphing strategy. The shape of the 3D circuits further transformed under wet air stream (Supplementary Fig. 21i).
